# Hedgehog-Interacting Protein is a multimodal antagonist of Hedgehog signalling

**DOI:** 10.1038/s41467-021-27475-2

**Published:** 2021-12-09

**Authors:** Samuel C. Griffiths, Rebekka A. Schwab, Kamel El Omari, Benjamin Bishop, Ellen J. Iverson, Tomas Malinauskas, Ramin Dubey, Mingxing Qian, Douglas F. Covey, Robert J. C. Gilbert, Rajat Rohatgi, Christian Siebold

**Affiliations:** 1 Division of Structural Biology, Wellcome Centre for Human Genetics, University of Oxford, Oxford, UK; 2 Science Division, Diamond Light Source, Harwell Science and Innovation Campus, Didcot, UK; 3 Departments of Biochemistry and Medicine, Stanford University School of Medicine, Stanford, CA USA; 4 Department of Developmental Biology, Washington University School of Medicine, St. Louis, MI USA; 5 Present Address: Evotec (UK) Ltd., Milton Park, Abingdon, UK

**Keywords:** Sterols, Membrane proteins, X-ray crystallography, Extracellular signalling molecules, Morphogen signalling

## Abstract

Hedgehog (HH) morphogen signalling, crucial for cell growth and tissue patterning in animals, is initiated by the binding of dually lipidated HH ligands to cell surface receptors. Hedgehog-Interacting Protein (HHIP), the only reported secreted inhibitor of Sonic Hedgehog (SHH) signalling, binds directly to SHH with high nanomolar affinity, sequestering SHH. Here, we report the structure of the HHIP N-terminal domain (HHIP-N) in complex with a glycosaminoglycan (GAG). HHIP-N displays a unique bipartite fold with a GAG-binding domain alongside a Cysteine Rich Domain (CRD). We show that HHIP-N is required to convey full HHIP inhibitory function, likely by interacting with the cholesterol moiety covalently linked to HH ligands, thereby preventing this SHH-attached cholesterol from binding to the HH receptor Patched (PTCH1). We also present the structure of the HHIP C-terminal domain in complex with the GAG heparin. Heparin can bind to both HHIP-N and HHIP-C, thereby inducing clustering at the cell surface and generating a high-avidity platform for SHH sequestration and inhibition. Our data suggest a multimodal mechanism, in which HHIP can bind two specific sites on the SHH morphogen, alongside multiple GAG interactions, to inhibit SHH signalling.

## Abstract (web summary)

Hedgehog-Interacting Protein (HHIP) is the only reported secreted inhibitor of Sonic Hedgehog (SHH) signalling. Here, the authors report structures of the HHIP N- and C-terminal domains, both in complexes with glycosaminoglycans, providing insights into the molecular basis for SHH sequestration and inhibition.

## Introduction

The Hedgehog (HH) morphogen pathway fulfils crucial functions in growth and morphogenesis, whilst dysregulation leads to developmental disorders and cancer^1–4^. The secreted N-terminal domain of SHH (ShhN) is generated from a 45 kDa precursor, undergoing intein-based cleavage to couple an esterified cholesterol molecule to the C-terminus^5^. A subsequent step involves the N-terminal attachment of a palmitoyl moiety to produce the fully active lipid-modified signalling ligand (palmitoylated and cholesteroylated ShhN; pShhNc)^6^. Signalling is activated by binding of pShhNc to the extracellular domains of transmembrane protein PTCH1. Recent structural studies show that PTCH1 and pShhNc form a 2:1 complex, with one molecule of PTCH1 engaging pShhNc at a conserved high-affinity interface involving the conserved SHH zinc- and calcium-binding sites (“protein-protein interface”), and the other at the terminal SHH-palmitoyl and -cholesteryl moieties (“lipid interface”)^7–12^. The PTCH1:pShhNc interaction releases inhibition of the G-protein coupled receptor Smoothened (SMO), which ultimately results in activation of target genes via the GLI transcription factors^2^. When no HH ligand is present, PTCH1 constitutively inhibits SMO signalling, potentially by preventing access to cholesterol or a similar sterol molecule^10,13–16^.

**Fig. 1:**
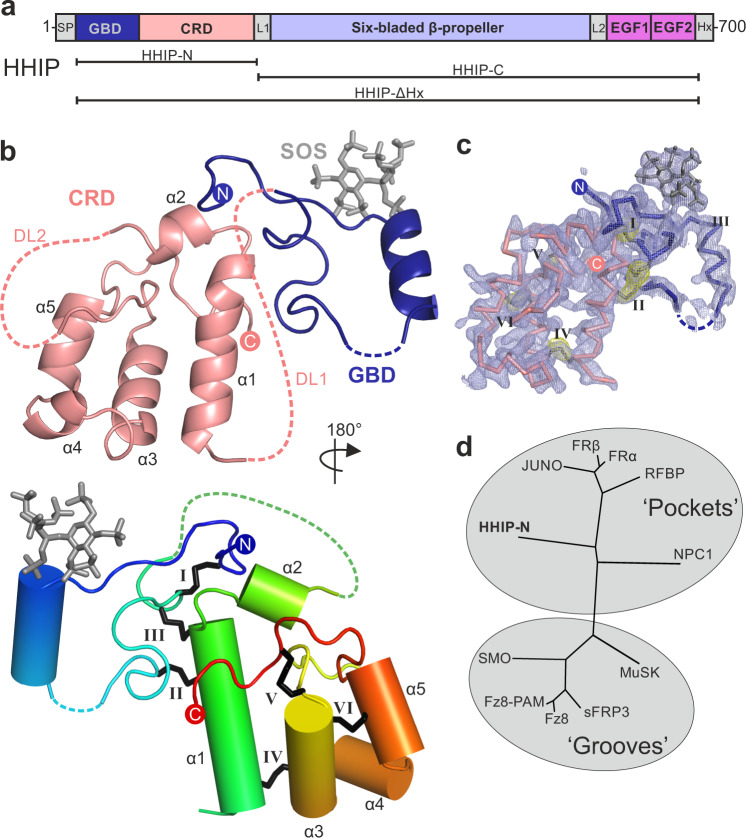
The structure of HHIP-N. **a** HHIP domain organisation and construct design. **b** Cartoon representation of HHIP-N in complex with SOS. In the top panel, the CRD region is depicted in salmon and the N-terminal GAG-binding domain in dark blue. Two disordered loops (DL1 and DL2) are displayed as dashed salmon lines. In the lower panel, the HHIP-N:SOS complex is shown in rainbow colouring (blue: N-terminus, red: C-terminus). The CRD helices are labelled and disulphide bonds are numbered using Roman numerals. **c** Maps calculated from final HHIP-N model. The 2Fo-Fc map is shown in blue, contoured at 1σ. An anomalous difference map calculated from S-SAD data used for phasing is displayed as a yellow mesh contoured at 4σ. Corresponding disulphide bonds are numbered using Roman numerals. **d** Structural phylogenetic analysis of CRDs (see Supplementary Table 2).

Extracellular distribution of pShhNc is key to the activation of correct signalling responses. This is controlled by a combination of co-receptor signalling^17^, the glycosaminoglycan (GAG) chains of heparan sulphate proteoglycans (HSPGs)^18,19^ and the assembly of pShhNc into multimers^20,21^. The HH pathway is modular, with several other essential cell surface receptors alongside PTCH1. For example, the immunoglobulin superfamily members CDO and BOC bind directly to SHH via the conserved interface involving the pShhNc metal-binding sites^22–25^. The metal-binding sites are also crucial for SHH interactions with the vertebrate-specific HH antagonist Hedgehog-Interacting Protein (HHIP)^26,27^. HHIP is the only secreted inhibitor of HH signalling^28–30^, essential for the development of the lung^31^, cartilage^32^ and brain^33^. HHIP downregulation is associated with HH-dependent tumourigenesis^34^ and variants at the *HHIP* locus are linked to Chronic Obstructive Pulmonary Disease (COPD), one of the most common devastating lung diseases in humans^35^. HHIP is composed of an N-terminal domain (HHIP-N) that shows weak sequence homology to the cysteine-rich domain (CRD) superfamily^36^, typically involved in small molecule-binding (Fig. 1a). The C-terminal domain of HHIP is composed of a β-propeller and two EGF repeats (HHIP-C). We and others previously determined structures of HHIP-C in complex with human HH ligands ShhN and DhhN, respectively^26,27^. HHIP utilises a loop inserted into blade 3 of its β-propeller to bind to the HH metal-binding site by directly coordinating the Zn ion. This suggests that HHIP inhibits HH function by sequestering the HH morphogen, and acts as a decoy receptor. Our recent study^28^ identified a role for HHIP—high affinity interaction with the GAG chains of heparan sulphate proteoglycans (HSPGs), as well as uncovering a cluster of residues in HHIP-N involved in this process. However, the role of the HHIP-N CRD and its potential small molecule binding properties remained elusive.

In this work, we use a combination of structural, biophysical and cellular studies to characterise the HHIP N-terminal region, revealing an unexpected GAG binding domain and a CRD with a small molecule-binding fold. We show that HHIP-N is necessary to convey they full signal inhibition by HHIP in response to pShhNc, and that the purified CRD binds to a mimic of the cholesteroylated HH C-terminus. Importantly, we also identify and structurally characterise two distinct GAG binding sites within HHIP-N and HHIP-C, respectively, and show that HHIP-C assembles into large HHIP-GAG oligomers. Our results reveal that HHIP uses a modular mechanism for SHH inhibition. HHIP targets both the SHH metal-binding and lipid-modification sites recognised by PTCH1, while potentially staying localized on the cell surface via HHIP-GAG interactions.

## Results

### Structure of the HHIP N-terminal domain reveals a CRD fold

We expressed the N-terminal domain of HHIP (HHIP-N) using mammalian expression in HEK293T cells^37^ (Fig. 1a). Purified HHIP-N was crystallised in the presence of the GAG mimic sucrose octasulphate (SOS). The structure of the HHIP-N:SOS complex was determined using the single anomalous dispersion (SAD) method from native sulphur atoms. A multi-crystal approach was taken in which 24 data sets were collected from 8 isomorphous crystals, utilising both mini-kappa and inverse beam strategies to maximise the observed anomalous signal (Supplementary Table 1, Supplementary Fig. 1a–d)^38,39^. This allowed us to determine the structure of HHIP-N in complex with SOS at a resolution of 2.7 Å, with one molecule in the crystallographic asymmetric unit. HHIP-N possesses an elongated globular fold with a unique N-terminal GAG-binding domain (GBD) and a C-terminal CRD (Fig. 1b). HHIP-N is stabilised by a total of 6 disulphide bonds. The GBD of HHIP-N binds SOS, and contains a single helix and flanking loop regions (Fig. 1b, lower panel) that are stabilised by one intra-domain disulphide bond (I: C39-78) and two inter-domain disulphide bonds with helix α1 of the CRD (II: C69-C112 and III: C79-115). The HHIP-N CRD is composed of 5 helices (α1-α5) stabilised by three intra-CRD disulphide bonds (IV: C103-C152, V: C141-179 and VI: C145-168). All disulphide bonds were identified as individual sites in the anomalous difference map, and treatment of these as ‘super-sulphurs’ was vital for substructure determination and structure solution (Fig. 1c)^40^.

**Fig. 2:**
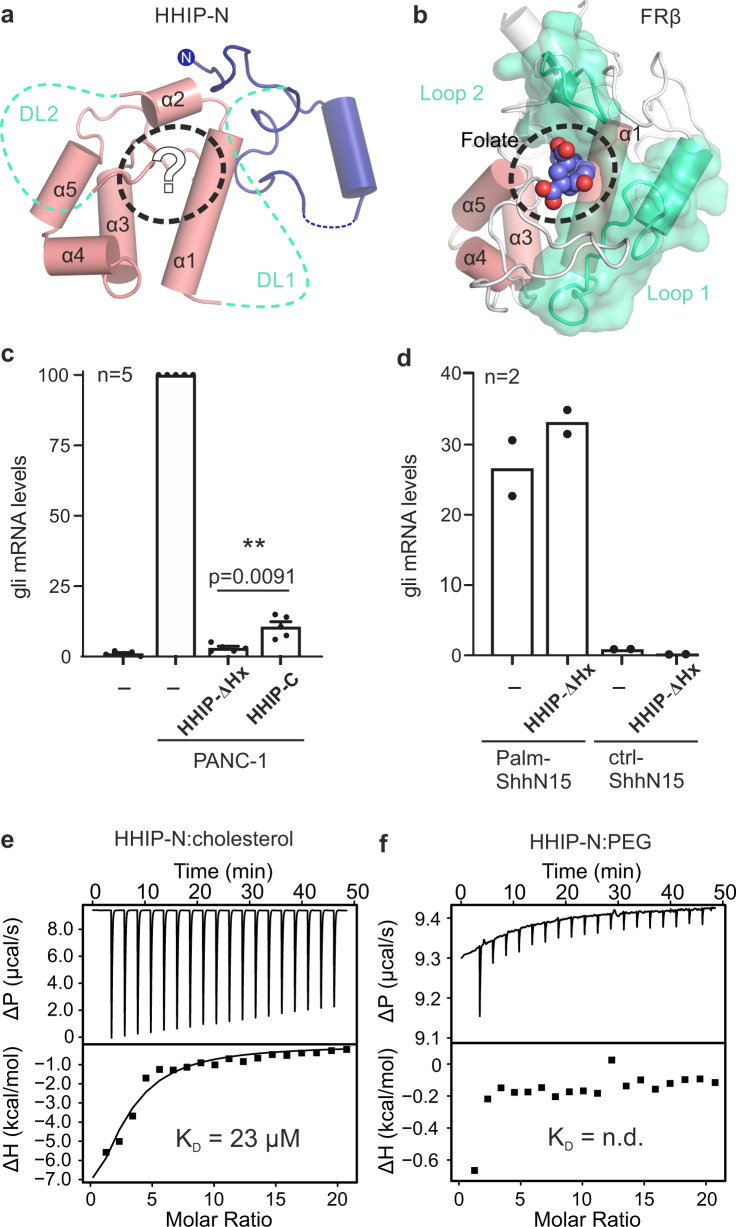
HHIP-N CRD contributes to HH signalling inhibition. **a**, **b** Structural homology of HHIP-N with CRDs suggests small molecule interaction. HHIP-N (**a**) is shown, with missing loops DL1 and DL2 displayed as green dashed lines and helices are annotated α1-5. A potential ligand-binding pocket is outlined with a dashed circle. FRβ (**b**) binds a folate molecule (spheres, circled), utilising loops structurally analogous to HHIP-N DL1 and DL2 (coloured green). Helices are annotated α1-5 as in HHIP-N. **c** RT-PCR assay from NIH/3T3 cells co-cultured with PANC-1 cells expressing pShhNc to quantify Hh signalling in the presence of HHIP constructs. Relative levels of Gli1 mRNA were quantified and normalised from 5 independent experiments and displayed as mean values ± SEM, with statistical significance calculated using a two-tailed, paired t-test with *p* = 0.0091. **d** HH signalling assay to assess HHIP-∆Hx inhibition of pathway activation in response to a palmitoylated N-terminal pShhNc peptide. **e** Raw ITC (upper panel) and binding isotherm (lower panel) for titration of PEG-cholesterol into HHIP-N. **f** Raw ITC (upper panel) and binding isotherm (lower panel) for titration of unconjugated PEG200 into HHIP-N. Source data are available as Source Data file.

CRDs can bind to various small molecules, including folate (folate receptors; FRα/β)^41^ and riboflavin (riboflavin-binding protein; RFBP)^42^, as well as lipids and sterols, such as cholesterol (SMO, NPC1)^43,44^ and palmitoleic acid (Frizzled; Fz)^45^. Previously, we performed an evolutionary structural analysis to classify known CRDs into two distinct sub-families, containing ‘pockets’ or ‘grooves’ as ligand-binding elements^46^. In this context, a ‘pocket’ refers to a CRD in which the ligand-binding site consists of loops that fold over a deep cavity inaccessible to solvent, whereas a ‘groove’ comprises a shallow and elongated solvent-accessible binding cleft, which does not undergo major conformational changes upon ligand binding. We have now included our HHIP-N structure in this analysis and classified it as a member of the pocket-type sub-family, with related structural homologues in the CRDs of NPC1, RFBP and FRα/β (Fig. 1d, Supplementary Table 2). Additionally, this analysis identifies sperm-egg fusion protein JUNO as being a pocket-type CRD which has diverged from typical folate receptors^47^. The HHIP-N CRD comprises a structural ‘scaffold’ composed of helices a1, a3 and a5 that are stabilised by disulphide bridges, a feature common to all CRD family members (Fig. 1b, Supplementary Fig. 2). In our HHIP-N structure, we observed two stretches of disordered residues, which link the N-terminal domain and CRD helix α1 (DL1), and the loop regions between helices α2 and α3 (DL2), respectively (Fig. 2a). In FRβ, the equivalent regions form the majority of a folate-binding pocket (Fig. 2b), suggesting that DL1 and DL2 very likely occupy a similar region. The modes of ligand binding by pocket-type CRDs are illustrated in Supplementary Fig. 3b, c and Supplementary Fig. 3f, while ligand binding by groove-type CRDs is detailed in Supplementary Fig. 3d-e. Helices analogous to HHIP-N α1-5 form an equivalent ‘scaffold’ in the pocket-type CRDs NPC1, RFBP and JUNO, within which ligand-binding loops are positioned for small molecule binding. A ligand-binding loop comparable to HHIP-N DL1 is also present in the groove-type CRDs of Fz8 and SMO (Supplementary Fig. 3d, e). This structural and evolutionary analysis suggests that loops DL1 and DL2 could potentially form a binding pocket for a physiologically-relevant small molecule ligand within the HHIP-N CRD region.

Next, we tested the function of HHIP-N in a SHH signalling assay, based on measuring the mRNA levels of the HH target gene Gli1 in NIH/3T3 cells. We co-cultured PANC-1 human pancreatic ductal adenocarcinoma cells expressing pShhNc^48^ to activate the HH pathway. The purified full-length HHIP extracellular domain only lacking the C-terminal helix (residues 39-670, HHIP-∆Hx, Fig. 1a) was able to inhibit HH signalling more efficiently than HHIP-C (Fig. 2c), which lacks HHIP-N but contains the high-affinity SHH binding site^26,27^. We next asked whether the interaction of HHIP-N with the SHH lipid modifications could account for this increase of inhibition, given the structural similarities with small molecule-binding CRDs. For this, we activated the HH pathway with an N-terminal palmitoylated-SHH peptide (Palm-ShhN15)^49^, which does not interact with HHIP-C. In this context, HHIP-∆Hx was unable to inhibit signalling (Fig. 2d), excluding a role of the SHH N-terminal palmitoyl moiety in HHIP-N binding. We recently showed that the SHH C-terminal cholesterol moiety is important for SHH-PTCH1 interactions and activation of HH signalling^12^. Using a similar isothermal titration calorimetry (ITC) assay, we tested whether a PEGylated cholesterol molecule (mimicking the C-terminus of pShhNc) binds to HHIP. PEG-cholesterol bound specifically to HHIP-N (K_D_ = 23 µM) (Fig. 2e), whereas free PEG did not bind to HHIP-N (Fig. 2f). Due to the high heat of dilution when PEG-cholesterol is titrated into buffer, a series of controls were conducted (Supplementary Fig. 4). Thermodynamic signature plots for HHIP-N compare favourably with those obtained for the recently published interaction of PEG-cholesterol with the canonical Hh receptor PTCH1^12^, revealing a high enthalpic contribution to ∆G suggesting a hydrophobic interaction^50^. This supports that the interaction between PEG-cholesterol and HHIPN is specific. Taken together, the enhancement of HH signalling inhibition by HHIP in the presence of HHIP-N, combined with structural homology to small molecule-binding pocket-type CRDs and observed binding to PEG-cholesterol suggests an interaction between HHIP-N and HH-linked cholesterol. Hence, we propose a model whereby HHIP binds to SHH in a multimodal manner, utilising the SHH metal-binding sites for HHIP-C binding and potentially targeting the cholesterol attachment site for HHIP-N binding. Such an arrangement could be accommodated in a 1:1 SHH-HHIP complex, as HHIP-N and HHIP-C are separated by a 25-residue long, likely flexible linker that could position the SHH-linked cholesterol to interact with HHIP-N and the SHH-metal binding site to interact with HHIP-C within the same molecule (Supplementary Fig. 5).

### The HHIP N-terminal domain contains a GAG-binding domain

**Fig. 3:**
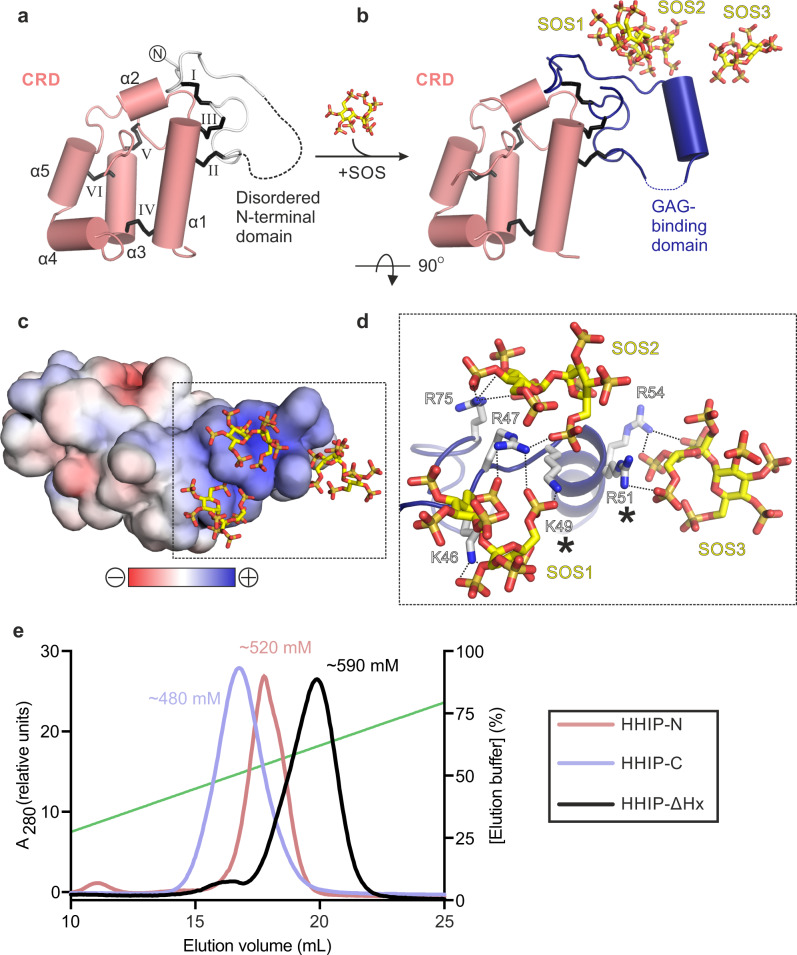
HHIP-N:GAG interactions. **a**, **b** Comparison of the apo HHIP-N structure (**a**) and HHIP-N:SOS complex. Colour coding is as in Fig. 1b. For HHIP-N:apo, the disordered GAG-binding domain is shown as a dashed line. In the HHIP-N:SOS complex three SOS molecules interact with the GAG-binding domain. **c** Electrostatic surface potential of the HHIP-N:SOS complex, displayed from −10 kT/e to +10 kT/e. **d** Close-up view of HHIP-N:SOS binding site showing the region marked in C. Hydrogen bonds are shown as dashed lines (**c**, **d** rotated 90^°^ around the x-axis relative to B). Mutations previously observed to decrease GAG-binding affinity and cause dysfunction of HHIP are marked with asterisks^28^. **e** Heparin affinity chromatography of different HHIP constructs eluted from a linear gradient of NaCl from 20 to 1000 mM (green line). Traces corresponding to different constructs are coloured according to the accompanying legend (inset).

To shed light onto the previously reported GAG-binding properties of HHIP-N^28^, we determined structures of apo and SOS-bound HHIP-N to resolution of 2.6 Å and 2.7 Å, respectively (Supplementary Table 1). In the apo HHIP-N structure, the N-terminal GAG-binding domain is disordered, while the CRD region is inherently structured (Fig. 3a). This suggests that interaction of HHIP-N with GAG molecules triggers a transition to a folded state, forming an α-helix between residues 50 and 58. Each HHIP-N molecule interacts with 3 SOS molecules (Fig. 3b, Supplementary Fig. 6). SOS is bound to a positively charged surface localised to the HHIP-N N-terminal domain (Fig. 3c), forming electrostatic interactions with a cluster of 6 basic residues and burying a total surface area of 594 Å^2^ (Fig. 3d). Only Arg-47 forms hydrogen bonds with more than one SOS molecule; Arg-51 and Arg-54 are the only residues within the N-terminal α-helical residues 50–58 to contact a SOS molecule. Mutagenesis of SOS-interacting basic HHIP residues (marked with asterisks in Fig. 3d) were previously observed to weaken the interaction between HHIP and heparin, validating our observed HHIP-N:SOS interface^28^. The N-terminal domain of HHIP-N is different compared to other structurally characterised CRDs, having evolved a GAG-binding function in a discrete domain alongside a conserved small molecule-binding fold. Additionally, we report structural insights into the major secondary structural rearrangements of HHIP-N, a GAG-binding protein, upon sugar coordination.

### Structure of the HHIP C-terminal domain in complex with heparin

Our previous analysis of HHIP:GAG interactions identified multiple GAG-binding motifs in HHIP, since a construct that contains a deletion of HHIP-N was still able to bind to heparin^27^. To decipher the GAG-binding properties of HHIP, we conducted binding assays using three domain deletion constructs (Fig. 1a). First, we tested GAG binding using heparin affinity chromatography (Fig. 3e). Both HHIP-C and HHIP-N show binding to a heparin column, albeit with weaker affinity when compared to HHIP-∆Hx. Furthermore, HHIP-N, HHIP-C and HHIP-∆Hx are all cell surface-associated at physiological pH and ionic strength (Supplementary Fig. 7). Taken together, our analysis suggests that both HHIP-N and HHIP-C contribute to the observed affinity for GAG chains.

**Fig. 4:**
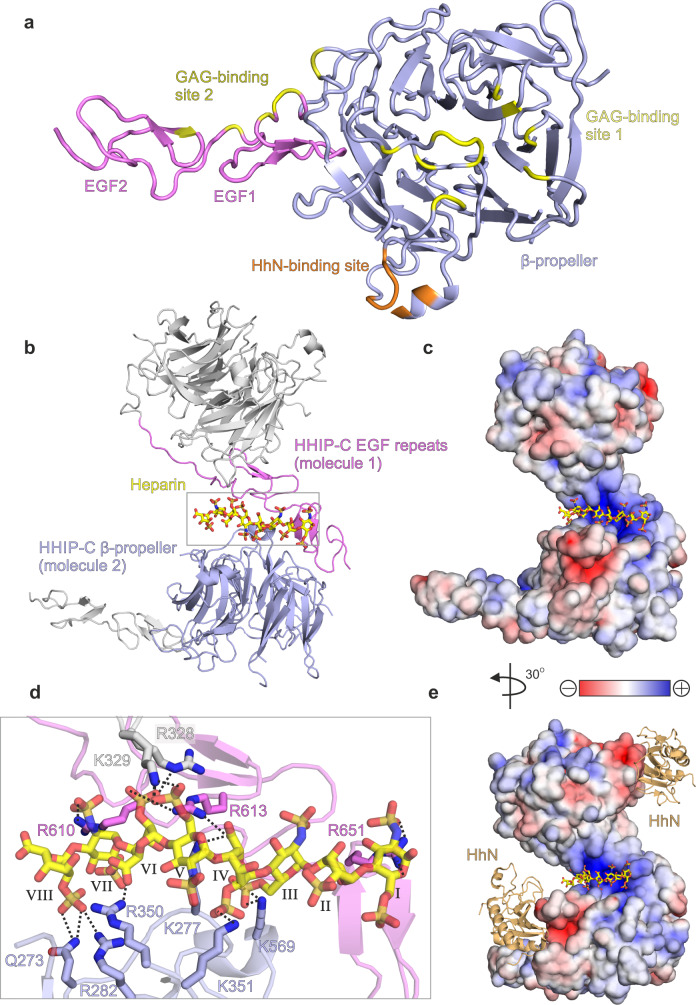
The structure of the HHIP-C:heparin complex. **a** Cartoon representation of the HHIP-C monomer structure with the β-propeller domain in slate, the EGF repeats in magenta, the GAG-binding sites in yellow and the HhN-binding site in orange. **b** Arrangement of the two HHIP-C molecules (cartoon representation) around a centrally-coordinated heparin molecule (stick representation). **c** Close-up view of the boxed region in B, displaying side chain:heparin interactions. Hydrogen bonds are displayed as black dashed lines and sugar residues are numbered using roman numerals (odd, N,O6-disulphoglucosamine; even, O2-Sulphoiduronic acid). **d** Electrostatic surface potential shown from red (−8 kT/e) to blue (+8 kT/e) with heparin chain shown as sticks. **e** Comparison of heparin (yellow sticks) and HhN (orange cartoon) interaction sites on HHIP-C electrostatic surface.

To structurally characterise HHIP-C:GAG interactions, we determined the 2.7 Å resolution crystal structure of HHIP-C in complex with a 30-mer heparin molecule (Supplementary Table 1, Supplementary Fig. 8a). HHIP-C comprises an N-terminal 6-bladed β-propeller and 2 C-terminal EGF repeats, previously identified in the complex of HHIP-C with HhN ligands^26,27^. The heparin chain contacts two separate clusters of HHIP surface residues (GAG ‘site1’ and GAG ‘site 2’) at either end of the molecule (Fig. 4a, Supplementary Fig. 8). In this arrangement, a HHIP-C anti-parallel dimer coordinates one single heparin chain (Fig. 4b) by using a central positively-charged region formed by residues from both HHIP protomers (Fig. 4c). The HHIP-C chains in the dimer are essentially identical (r.m.s.d. of 0.21 Å for 322 equivalent Cα atoms), and show little structural difference to previously published HHIP-C structures (e.g. r.m.s.d. of 0.31 Å for 331 equivalent Cα atoms, PDB ID. 2WFT^26^). The heparin backbone displays a right-handed helical structure with roughly 4 sugars per turn, in agreement with previous structural studies^51,52^. A total of 8 monosaccharides are resolved in the structure, running from a reducing (O1; sugar I) to a non-reducing end (O4; sugar VIII) and forming hydrogen bonds with several polar side chains (Fig. 4d). We also determined the crystal structure of a HHIP-C:SOS complex, which exhibits the equivalent anti-parallel, dimeric HHIP arrangement bound to two SOS molecules coordinated at the same site compared to heparin (Supplementary Table 1, Supplementary Figs. 8b, 9 and 10). In summary, our HHIP-C crystal structures in complex with GAG molecules identify two discrete GAG-binding sites on the surface of HHIP-C, which are distinct and non-overlapping with the HhN-binding interface (Fig. 4e), thus suggesting that both GAG- and HhN-binding can occur simultaneously.

### GAG interactions control oligomerisation of HHIP

**Fig. 5:**
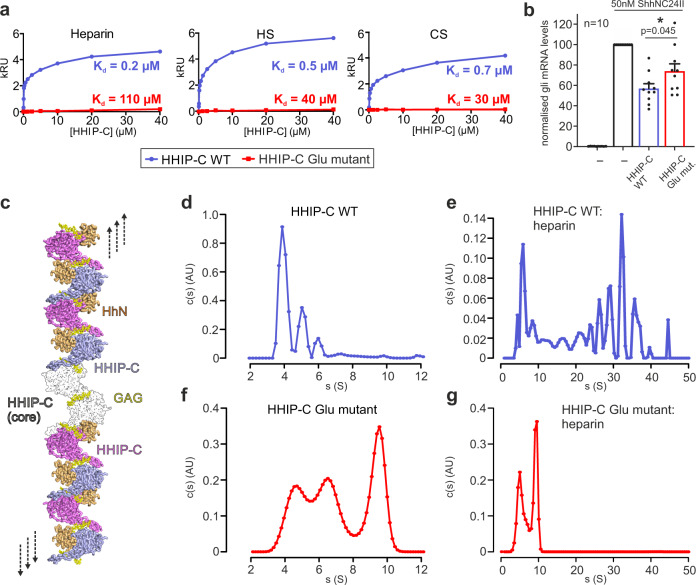
Analysis of HHIP-C:GAG interactions and oligomerisation. **a** Comparative SPR curves for HHIP-C WT (light blue) and HHIP-C Glu mutant (red) binding to heparin, heparan sulphate (HS) and chondroitin sulphate (CS); **b** Quantification of HH pathway inhibition with the addition of HHIP-C WT or HHIP-C Glu mutant. Relative levels of Gli1 mRNA were quantified from 10 independent experiments, with statistical significance calculated using a two-tailed, paired t-test with *p* = 0.045; Data are presented as mean values ± SEM. **c** Model for cell surface HHIP-C oligomeric assembly. GAG chains (represented by heparin, yellow) stabilise a core anti-parallel HHIP-C dimer (surface; black and white) and can then recruit further HHIP-C chains (light blue, violet) to form long oligomeric chains. This facilitates binding and antagonism of multiple HhN ligands (orange). **d**, **e** AUC experiments to assess the oligomerisation of HHIP in solution, comprising HHIP-C (**d**) and HHIP-C:heparin 30-mer complex (**e**); **f**, **g** As in (**c**, **d**), comprising HHIP-C Glu mutant (**e**) and HHIP-C Glu mutant mixed with 30-mer heparin (**f**). Source data are available as Source Data file.

Our previous study on HHIP:GAG interactions identified low micromolar affinities for the interactions between HHIP-C and the GAGs heparin, heparan sulphate (HS) and chondroitin sulphate (CS)^28^. To experimentally validate our HHIP-C:GAG complex structures, we generated a HHIP mutant in which 6 positively-charged residues involved in GAG recognition were mutated to glutamate (K277E/R328E/R350E/K569E/R610E/R613E, HHIP-C Glu mutant; Fig. 4d) and analysed binding to GAGs. Using surface plasmon resonance (SPR) with GAGs immobilised on the chip, we observed that the HHIP-C Glu mutant almost completely abolishes binding to heparin, HS and CS, when compared to wild-type (HHIP-C WT) (Fig. 5a). Furthermore, we analysed our observed HHIP-C:GAG interfaces by a combination of mutagenesis and heparin affinity chromatography. In addition to the HHIP-C Glu mutant, we generated glycosylation mutants (Supplementary Fig. 10a). N-linked glycans were inserted into the GAG-interacting surfaces of either the HHIP β-propeller (‘ΔGAG site 1’) or EGF repeats (‘ΔGAG site 2’), as well as combination of both (‘ΔGAG sites 1 + 2’). As expected, all mutants showed reduced heparin binding affinity relative to HHIP-C WT (Supplementary Fig. 11b). Next, we tested the effect of HHIP-C WT or HHIP-C Glu mutant in SHH signalling assays using NIH/3T3 cells (Fig. 5b). We observed that the HHIP-C Glu mutant showed significantly less efficient SHH inhibition. To further probe whether this effect results from loss of GAG-binding, we knocked out EXTL3, a key enzyme in the biosynthesis of cell-surface heparan sulphate (HS) chains (Supplementary Fig. 12a–d)^53^. We confirmed that single *Extl3*^−/−^ NIH/3T3 clones lack surface HS chains by showing markedly reduced binding to a scFv antibody (HS20) that is known to recognize multiple HS chains^54,55^ (Supplementary Fig. 12e). These *Extl3*^−/−^ clones were responsive to SHH, showing that the machinery required to receive and respond to ligands was unaffected by the loss of HS chains (Supplementary Fig. 12e). Compared to their individual level of SHH activation, wildtype HHIP was a less effective inhibitor in both knock-out cell lines than in their parental cell line. Even more importantly, both HHIP wildtype and Glu mutant inhibited the HH response in the HS-deficient cells to similar degrees, in contrast to the parental cell line where Glu mutant HHIP was a less effective inhibitor (Supplementary Fig. 12e). This demonstrates that the interaction between HHIP-C and GAGs are critical for maximal HH pathway inhibition.

Our HHIP-C:heparin complex structure also suggests that GAGs can mediate the formation of long oligomeric chains of HHIP (Fig. 5c). We observe two HHIP oligomerisation interfaces - an anti-parallel ‘head-to-tail’ heparin-bound HHIP-C dimer (Supplementary Fig. 13a) with a total buried surface area of 603 Å^2^ (Supplementary Fig. 13a), and a ‘head-to-head’ dimer with a buried surface area of 981 Å^2^ (Supplementary Fig. 10b) in the crystal. The ‘head-to-tail’ dimer interface is smaller than physiological dimer interfaces and is likely stabilised by the heparin molecule (PISA Δ^i^G *P*-value = 0.41, shape complementarity score = 0.75)^45,46^, while the larger ‘head-to-head’ interface is more likely to be physiological (PISA: Δ^i^G *P*-value = 0.43, shape complementarity score: 0.52). Previous studies using multi-angle light scattering (MALS) showed that the apo HHIP-C is monomeric at a concentration of approximately 10 μM^26^. To test whether oligomers observed in the crystal structure exist in solution at higher concentrations (potentially mimicking the local concentrations at the cell membrane), we performed sedimentation velocity analytical ultracentrifugation experiments (AUC). This revealed a predominantly monomeric apo HHIP-C population (4 S, 53 kDa), with minor populations of dimers (5 S, ~100 kDa) and trimers (6 S, ~150 kDa) also observed (Fig. 5d). As expected, addition of the heparin 30-mer triggers a drastic shift to increased sedimentation rates, and causing the sedimentation of an array of higher-order oligomeric species from 50 kDa up to 6 MDa (4 S–50 S; Fig. 5e). Higher-order oligomer formation in response to the addition of heparin is completely ablated in the HHIP-C Glu mutant (Fig. 5f–g), consistent with GAG-binding induced oligomerisation of HHIP. Taken together, our biophysical and cellular data suggest that HHIP:GAG complexation leads to HHIP clustering, and these assemblies might fine-tune inhibition of the HH signal.

HHIP-N can also bind GAGs with high affinity and despite crystallising as a monomer, forms weak dimers in solution when analysed using MALS (Supplementary Fig. 14a). Interestingly, apo HHIP-N is predominantly monomeric in solution up to 200 µM when studied by AUC (Supplementary Fig. 14b), and can also form oligomers in the presence of heparin (up to a size consistent with tetramers, as shown from c(s, f/f0) plots to analyse molecular weights in solution; Supplementary Fig. 14c). This is consistent with our crystal packing analysis (Supplementary Fig. 4), and suggests that GAGs stabilise a contact between HHIP-N GAG-binding domains in solution to assist oligomerisation (Supplementary Fig. 14d). In conclusion, HHIP is regulated through both protein:protein- and protein:GAG-mediated oligomerisation at several different sites, that can be linked to dynamic modulation of HH signalling.

## Discussion

**Fig. 6:**
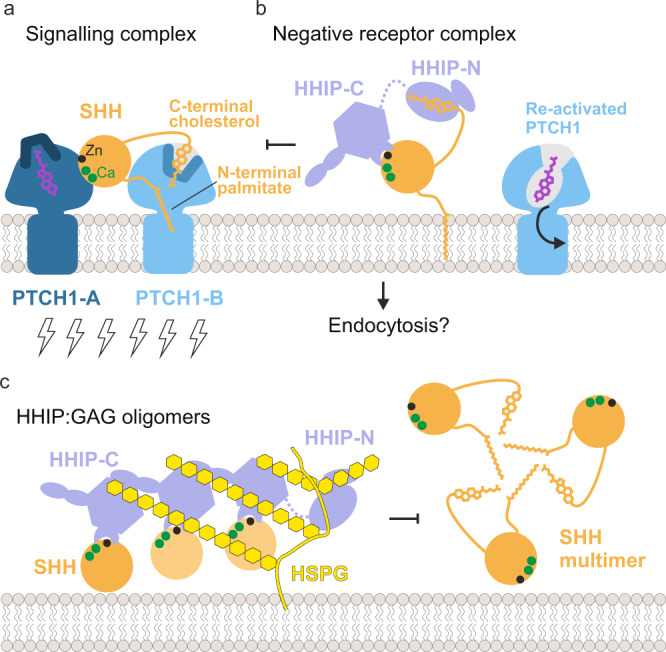
Models of extracellular HH signalling antagonism by HHIP. **a** A 2:1 interaction of PTCH1:pShhNc represents the active signalling complex. The pShhNc lipid appendages insert to seal channels in PTCH1-B. **b** HHIP-C targets the conserved high-affinity metal ion-binding site on the pShhNc surface (‘protein-protein interaction’), competing with binding from PTCH1-A and other co-receptors, whilst HHIP-N is able to contribute to this by targeting the C-terminal cholesterol moiety, possibly re-establishing PTCH1 cholesterol transport ability. It is also of note that HH signalling is activated via SMO CRD-cholesterol interaction, and competition of this by the HHIP-N CRD represents a possible additional mode of inhibition. Additionally, HHIP-mediated receptor endocytosis may act to remove SHH from the cell surface. **c** Cell surface GAG-mediated clustering represents a second modality for HH regulation by HHIP. The avidity of HHIP is increased by cluster formation at the proteoglycan layer, similar to how SHH lipoprotein multimers act to increase local morphogen concentration. The GAG-binding site in HHIP-N is also important for this process.

The function of the N-terminal domain of HHIP has remained a long-standing mystery. Previous work in our laboratory and by others showed that HHIP-C binds to the metal-binding site of SHH with high (low nanomolar) affinity, and inhibits HH signalling by functioning as a decoy receptor^26^. Our structural, biophysical and cellular work reveals that HHIP-N contains a CRD, which acts as an additional module within HHIP to inhibit HH signalling. Moreover, HHIP-N has evolved a structured region engaged in cell surface heparan sulphate proteoglycan binding. Recent pioneering work on the PTCH1:pShhNc interaction has greatly enhanced our understanding of HH signal reception and transduction. PTCH1 and SHH interact in a 2:1 stoichiometry, with one PTCH1 molecule (PTCH1-A) binding to the high-affinity metal-binding site of SHH and the other PTCH1 molecule (PTCH1-B) grasping the two lipid modifications to form the full signalling complex (Fig. 6a). None of these interactions alone is sufficient to fully inactivate PTCH1 function in cellular assays^12^. This mechanism shows parallels to WNT morphogen signalling, in which WNT interacts with its receptor Frizzled (Fz) with both its covalently-linked palmitoleate (via the Fz-CRD, evolutionarily related to the HHIP-N CRD), and also via a protein-protein interface^45^. WNT inhibition is, in part, achieved by interactions with secreted Fz-related proteins (sFRPs) (via both palmitoleate- and protein-protein contacts), which act as secreted decoy receptors^56^. Our structural and functional analysis suggests that HHIP has potentially evolved a similar role, targeting the SHH cholesterol moiety (via HHIP-N) and the metal-binding site (via HHIP-C). Thus, a two-pronged engagement with both protein- and lipid-based interfaces seems to be a common theme in recognition of morphogens.

Insertion of the SHH palmitoyl and cholesteryl moieties into the ectodomain of PTCH1-B blocks a proposed conduit for cholesterol/sterol transport^57,58^. Shielding of the pShhNc cholesterol modification by HHIP-N would be an effective mechanism to release SHH-mediated inhibition and transport function of PTCH1. This is complemented by HHIP-C and PTCH1-A competing for the SHH metal-binding site, which also overlaps with the binding sites for co-receptors CDO, BOC and GAS1^17,22^. These two modes of signal antagonism constitute a fail-safe mechanism by which HHIP regulates HH signalling. HHIP-C binds to the SHH metal-containing site some 100-fold tighter compared to CDO^22,26^ and PTCH1^12^, and thus could outcompete both CDO and PTCH1 from this site. This could result in the formation of a ‘negative receptor complex,’ facilitating for example SHH endocytosis (Fig. 6b).

Various studies have identified HHIP as the only known secreted diffusible inhibitor of HH signalling^28,29,59^. We previously showed that HHIP secretion is dependent on interaction with GAGs, and that HHIP can bind to various types of GAGs^28^. Here, we have delineated the molecular basis for this interaction. Both HHIP-N and HHIP-C contain discrete GAG-binding sites, which combine to modulate cell surface affinity. Together, they constitute additional minor regulatory sites of HH signalling. Cell surface-attached GAGs such as HS and CS organise secreted proteins into gradients, varying local concentrations to enable graded signal activation^60^. Here, we show that GAGs are able to cluster HHIP-C into large assemblies, linked to the potency of HHIP-mediated HH inhibition (which is further potentiated by HHIP-N). In addition, the HHIP C-terminal helix (residues 671–700) that contributes to cell surface binding via formation of a predicted leucine zipper-type assembly^28^ may enhance clustering even further, combined with the dimerisation abilities observed for both HHIP-N and HHIP-C. HHIP clustering by GAGs at the cell surface generates a high-avidity platform for SHH binding. This platform might limit diffusion and loss of HHIP into extracellular space and position HHIP to inhibit SHH signalling at the cell surface (Fig. 6c). This process can be further regulated by SHH-mediated internalisation of HHIP^29^, which potentially can happen at both SHH producing or responding cells. From our structural analysis, we also note that there is a long flexible linker between HHIP-N and HHIP-C, which we previously described as being proteolytically sensitive^26^. Cleavage of this linker may regulate the overall cell surface affinity of HHIP in vivo. Taken together, our results indicate that HHIP regulation of HH signalling is dynamic, and inhibitory processes are modularised through distinct functionalities within the HHIP N- and C-terminal domains.

Aberrant expression of SHH has been linked to the initiation and progression of numerous cancers, and HH inhibitors targeting SMO are in the clinic against basal cell carcinomas^3,4^. The SHH-specific mouse antibody 5E1 binds tightly to the SHH metal-binding site, overlapping with HHIP^61^, and has been shown to inhibit SHH activity in vivo and to reduce tumour growth in a pancreatic cancer mouse model^62^. HHIP could be used in a similar way, working as an engineered biologic to inhibit HH signalling. Here, we provide the framework for the development of HHIP-based HH inhibitors that can specifically preserve or block the different SHH-receptor interaction sites, and this interaction mode can be further fine-tuned by the GAG-binding properties and resultant HHIP clustering. Various biomedically-important secreted signalling proteins are reported to undergo GAG-dependent clustering^63^, and drugging such mechanisms could present an unexploited avenue for therapeutic discovery.

## Methods

### Protein expression and purification

Constructs of human HHIP (UniProt ID: Q96QV1) comprising the N-terminal domain (HHIP-N, 39-209), C-terminal domain (HHIP-C, 213-670)^26^ and a full-length variant only lacking the C-terminal helix (HHIP-∆Hx; 39-670) were cloned in the pHLsec vector in frame with a C-terminal His_6_-tag^37^. Additionally, HHIP-∆Hx was designed to contain a stabilised linker between HHIP-N and HHIP-C, comprising 7 point mutations (R185A, K186A, R189A, K204A, R210A, K211A and K213A) (see Supplementary Table 3 for used primers). This was performed to prevent interdomain proteolysis, as was observed previously for HHIP-∆Hx constructs^26,27^. HHIP constructs were expressed by transient transfection in HEK293T cells following a similar procedure as described in^26^. Briefly, HEK293T cells were grown in expanded-surface polystyrene roller bottles (2125 cm^2^, Greiner Bio-One). Per roller bottle, a transfection cocktail was prepared by incubating 1 mL of 1 mg mL^−1^ 25 kDa branched polyethylenimine (Sigma Aldrich) with 0.5 mg plasmid DNA in 50 mL serum-free DMEM for 10 min at room temperature. Prior to addition of the cocktail to the cells, the mixture was supplemented with 1 µg mL^−1^ final concentration of the glycosylation inhibitor kifunensine^64^. Proteins were then expressed for 3–5 days in DMEM supplemented with 2% (v/v) FBS and 2 mM L-Glutamine/non-essential amino acids. Conditioned medium was dialysed against PBS and proteins were isolated via immobilised metal-affinity chromatography using a HisTrap HP^TM^ column (GE Healthcare). All proteins were subsequently purified further via size exclusion chromatography in a buffer of 10 mM HEPES pH 7.5, 150 mM NaCl. In the case of HHIP-N and HHIP-∆Hx, a further purification step utilising a HiTrap Heparin HP^TM^ (GE Healthcare) column was added to remove co-eluting degradation products.

### Heparin affinity chromatography

Purified HHIP constructs were loaded onto a 1 mL Heparin column (HiTrap Heparin HP: Life Technologies) equilibrated in 20 mM NaCl, 10 mM HEPES pH 7.5 (~3 mS/cm). Constructs were eluted over 20 column volumes using a linear gradient of elution buffer up to 1000 mM NaCl, 10 mM HEPES pH 7.5 (~85 mS/cm). The elution of HHIP constructs was followed via absorption at 280 nm. HHIP-N elutes at 520 mM NaCl, 10 mM HEPES pH 7.5 (~44 mS/cm), HHIP-C at 480 mM NaCl, 10 mM HEPES pH 7.5 (~41 mS/cm) and HHIP-ΔHx at 590 mM NaCl, 10 mM HEPES pH 7.5 (~50 mS/cm).

### Crystallisation and data collection

Prior to crystallisation trials, proteins were concentrated via ultrafiltration (HHIP-N, 6.8 mg mL^−1^; HHIP-C, 7.0 mg mL^−1^) and deglycosylated by addition of catalytic quantities of endoglycosidase F1^64^ at a ratio of 1:250 (w/w) (HHIP:EndoF1). For HHIP co-crystallisation with GAGs, either 10 mM SOS (Toronto Research Chemicals) or 1.5 mM 30-mer heparin (Iduron) was added to the concentrated protein. Nanolitre-scale crystallisation trials were performed using a Cartesian Technologies robot (100 nL protein plus 100 nL reservoir solution) in 96-well Greiner plates^65^. The HHIP-N:SOS complex was crystallised in 0.1 M imidazole/MES pH 6.5, 0.1 M carboxylic acids (0.02 M sodium formate, 0.02 M ammonium acetate, 0.02 M sodium citrate, 0.02 M sodium oxamate and 0.02 M potassium sodium tartrate), 10% w/v PEG 20000 and 20% v/v PEG MME 550; apo HHIP-N was crystallised in 0.1 M HEPES pH 7.0, 0.2 M ammonium chloride, 0.02 M hexamine cobalt (III) chloride, 0.002 M SOS and 20% w/v PEG 6000. The HHIP-C:heparin complex was crystallised in 0.1 M bis-Tris pH 6.5, 0.2 M sodium citrate and 20% w/v PEG 3350; the HHIP-C:SOS complex was crystallised in 0.1 M Tris/Bicine pH 8.5, 0.03 M magnesium chloride, 0.03 M calcium chloride, 10% w/v PEG 20 000 and 20% v/v PEG MME 550. All crystals of HHIP were grown at 20 ^°^C.

Diffraction data were collected at a temperature of 100 K with crystals mounted within a liquid N_2_ cryo-stream. Crystals were treated with an appropriate cryoprotectant supplemented with reservoir solution (HHIP-N:SOS, 15% glycerol; apo HHIP-N, 30% ethylene glycol; HHIP-C:heparin, 30% ethylene glycol; HHIP-C:SOS, 20% glycerol) and flash-cooled in liquid N_2_ prior to data collection. Data were collected at Diamond Light Source, UK on beamlines I03 (HHIP-C:heparin, λ = 0.97631 Å), I04 (HHIP-N:SOS; HHIP-C:SOS, λ = 0.97949 Å) and I24 (apo HHIP-N, λ = 0.96862 Å). Data collection of HHIP-N for experimental phasing by the Sulphur-Single Anomalous Dispersion (S-SAD) method was carried out at λ = 1.7712 Å. This wavelength was selected to maximise the observed anomalous signal without compromising the transmission of X-rays^66^. To counteract the low anomalous signal from S atoms at this wavelength, S-SAD data collection of HHIP-N:SOS crystals was performed using inverse beam (I03) and mini-kappa goniometry-based (I04) approaches^38,39^. A total of 24 datasets on 8 different crystals were collected. In all other cases, data were collected using the standard rotation method. Diffraction data were scaled and merged using *XIA2*^67–72^. The HHIP-C:heparin complex data was processed using the program *DIALS* in combination with *XIA2*^73^. In all cases, an inner shell with a CC_1/2_ of 0.30 was utilised in the selection of a high-resolution cut-off^74^.

### Structure solution

We determined the structure of the HHIP-N:SOS complex by S-SAD phasing. The sulphur substructure was determined using the HKL2MAP interface for the SHELX suite^75,76^. Using SHELXC, an anomalous signal to 4 Å resolution was observed, and searching for 6 sulphur sites as disulphides in SHELXD gave a solution with CC_weak_ and CC_all_ statistics of 23.8 and 42.4 respectively. These SHELXD substructure coordinates were used as input files for an initial round of phasing in *Phenix Autosol* and *Autobuild* pipelines^77,78^. This enabled determination of a partial HHIP-N structure, with 87 of 182 residues built (Supplementary Fig. 1a). This partial model was then used as input for Molecular Replacement-SAD in *Phaser*^79^. This allowed extension of the model to 98 residues placed in the asymmetric unit (Supplementary Fig. 1b). Manual building in *Coot*^80^ and initial refinement in *Refmac5*^81^ enabled the tracing of 110 residues, also resulting in visible electron density for a SOS molecule (Supplementary Fig. 1c). The HHIP-N SOS complex was refined using iterative cycles of refinement in BUSTER 2.10.3^82^ and *Phenix*^83^ (Supplementary Fig. 1d). Calculation of an anomalous difference map for the final HHIP-N:SOS structure identified 6 peaks at 4σ, corresponding to the 6 disulphide bonds (Supplementary Fig. 1e). The apo HHIP-N structure was solved by molecular replacement using the HHIP-N:SOS complex as a search model in *Phaser*^84^ and subsequently refined using *Phenix*^83^.

Both the HHIP-C:heparin and HHIP-C:SOS complexes were solved by molecular replacement using the HHIP-C apo-structure (PDB ID: 2WFT; residues 213-670)^26^ as a search model in *Phaser*^75^. The HHIP-C:heparin complex was refined using Buster 2.10.3^82^, and HHIP-C:SOS with *Phenix*^83^. For the HHIP-C:heparin complex, 8 sugars were accounted for by the electron density per asymmetric unit. Restraints were generated for both pyranose constituents (IDS, SGN) of heparin using the program *Privateer*^85^. For the HHIP-C:SOS complex, 2 SOS molecules were visible in the electron density per HHIP-C chain (2 HHIP-C chains per asymmetric unit).

### Structure analysis

Stereochemical properties were assessed using the MolProbity server^86^. Surface electrostatic potentials were generated using *APBS*^87^. Superpositions were calculated using *PYMOL* (www.pymol.org), which was also used to generate ray-traced images of protein structures for figures. Residues involved in interactions were identified using both the PDBSUM and PISA servers^88,89^. The solvent accessible radius was set to 1.4 Å for the representation of all protein surfaces. Structural evolutionary analysis of CRDs was performed using *SHP*^90,91^ and *PHYLIP*^92^ to assemble a phylogenetic tree (Supplementary Table 2). Structure-based sequence alignments of HHIP-N with evolutionarily-related CRDs were generated using *UCSF Chimera*^93^ and were prepared for publication using the program *Aline*^94^. Carbohydrate stereochemistry was validated with *Privateer*^85^.

### Analytical ultracentrifugation (AUC)

For AUC experiments HHIP-N and HHIP-C were dialysed into 10 mM HEPES pH 7.5, 120 mM NaCl. Experiments were performed at 20 °C using a Beckman Optima XL-I analytical ultracentrifuge (Beckman Instruments) with absorbance optics at 280 nm and interference optics. HHIP-N samples were spun at a concentration of 0.7 mg mL^−1^, alone and with the addition of 0.09 mM 30-mer heparin (dp30, Iduron). HHIP-C samples were spun at a concentration of 1 mg mL^−1^, alone and with the addition of 0.16 mM 30-mer heparin (dp30, Iduron). As a control, heparin was spun alone in a separate chamber at the concentration specified and analysed using interference optics. Samples were contained within 12 mm Epon sector-shaped two-channel centerpieces and spun at 128,794 × *g* (An60Ti rotor, Beckman Coulter Inc., CA), with 80 sample distribution scans taken in 6 min intervals, alongside interference optics. Absorbance data for scans 5–50 were analysed using the program SedFit for size-and-shape distributions c(*s*) and (c(*s,fr*), where *fr* is the frictional ratio and for a sphere *fr* = 1 and for other species *fr* > 1)^95^. This enables the plotting of contour plots of *c(s,M)*, where *M* is the weight of the protein. Due to the complex distributions observed in the case of HHIP-C:heparin, we present only c(*s*) distributions for HHIP-C experiments. For full clarity of solution molecular weights, c(*s, fr*) distributions were calculated for HHIP-N. In all cases, a partial specific volume value of 0.73 mL g^−1^ was used.

### Surface plasmon resonance

SPR experiments were performed using a Biacore T200 machine (GE Healthcare) in 10 mM HEPES pH 7.5, 120 mM NaCl, 0.05% (v/v) polysorbate 20, at 25 °C. Proteins were buffer exchanged into running buffer and concentrations were calculated from the absorbance at 280 nm using predicted molar extinction coefficient values. Heparin (Iduron; average molecular weight >9000 Da), heparan sulphate (HS) from porcine mucosa (Iduron) and chondroitin sulphate (CS) sodium salt from shark cartilage (Sigma) were biotinylated using EZ-link Biotin-LC-Hydrazide (Thermo Fisher Scientific) in a solution containing 17% (v/v) DMSO for 26 hours at 20 °C. GAGs were then extensively dialysed, first against water and then SPR running buffer (120 mM NaCl, 10 mM HEPES pH 7.5, 0.05 % v/v Tween 20), similar to a procedure described previously^96^. Biotinylated sugars were immobilised on CM5 sensor chips to which 3000 RU of streptavidin were covalently coupled^97^. After each binding experiment, the chip was regenerated with running buffer supplemented with 1.5 M NaCl at 30 μL min^−1^ for 120 s. In all experiments, the trace returned to baseline following regeneration. HHIP constructs were injected at a flow rate of 5 μL min^−1^. All data were analysed using SCRUBBER2 (Biologic) and GraphPad Prism Version 6.04 (GraphPad Software, La Jolla California USA). Best-fit binding curves were calculated using non-linear curve fitting of a one-site–total binding model (*Y* = *[R*_max_*X/(K_*D*_ + *X)]* + NS*X + *Background]*, where X is analyte concentration and the level of non-specific binding is assumed to be proportional to the analyte concentration; hence NS equals the slope of non-specific binding). The background value was set to zero as the data had been previously referenced. *R*_*max*_ and *K*_*D*_ values quoted are determined for the specific binding portion only.

### Isothermal titration calorimetry

Experiments were performed using a MicroCal PEAQ-ITC (Malvern) at 25 °C in 10 mM HEPES, pH 7.5, 150 mM NaCl and 3% isopropanol, with a differential power of 10 μcal s^−1^ and stirring at 750 rpm. Experiments consisted of an initial test injection of 0.4 μL, followed 150 s later by 18 injections of 2 μL, spaced 150 s apart. Owing to the low solubility of cholesterol, a PEG-cholesterol was used for affinity measurements. HHIPN was dialysed against 0.15 M NaCl, 10 mM HEPES and 3% isopropanol, final pH 7.5. Lyophilized PEG-cholesterol and PEG200 were resuspended in dialysis solution to a concentration of 1 mM. Protein concentrations were determined from the absorbance at 280 nm using calculated molar extinction coefficients. Cell concentrations of 9 μM HHIPN protein and syringe concentrations of 1 mM PEG-cholesterol or PEG200 were used for all experiments. Thermograms were integrated and corrected for heats of dilution using PEAQ-ITC analysis software (Malvern). Isotherms were fitted with the *A* + *B ⇌ AB* model, where cell and syringe concentrations and baselines of each experiment were fitted locally. All figures were prepared using PEAQ-ITC analysis software (Malvern).

### Immunofluorescence microscopy

HEK 293 T cells were seeded at a concentration of 10,000 cells mL^−1^ in poly-D-lysine coated 35 mm dishes (MatTek) and incubated at 37 °C, 5% CO_2_ for 18 h. Media was exchanged to reduce serum concentration from 10 to 2% and cells were transfected with 2 μg DNA of HA-tagged constructs using lipofectamine at a ratio of 1:2. 6 hours following transfection, cellular growth medium was further lowered to 0.5% serum and cells were further incubated for 2 days. Media was removed and cells were washed with PBS before fixation for 15 min with 3% para-formaldehyde and quenching in 0.3 M glycine for 3 min. Fixed cells were stored overnight at 4 °C in PBS. For the staining process, fixed cells were blocked for 10 min using 1% BSA/PBS, before HA tag probing using a Mouse HA Epitope Tag Antibody (Thermo Fisher) at 1 μg mL^−1^ in 1% BSA/PBS at 25 °C for 1 h. Cells were washed in PBS 3 times for 10 min, before incubation with an Alexa Fluor® 633-conjugated Goat anti-Mouse IgG antibody (λ_ex_: 633 nm; λ_em_: 647 nm) (Thermo Fisher) at a concentration of 2 μg mL^−1^ in 1% BSA/PBS for 1 h at 25 °C. Excess antibody was removed via washing 3 times in PBS for 10 min, with one final wash containing 0.5 μg mL^−1^ Hoechst 33342 nuclear stain (λ_ex_: 353 nm; λ_em_: 483 nm)^98^. Immunofluorescence was detected using a Leica TCS SP8 WLL Confocal SMD Microscope. Images were processed in Fiji^99^.

### Multiangle light scattering (MALS)

A total of 100 μL protein samples were injected onto an S200 10/30 column (GE Healthcare) equilibrated in a running buffer of 10 mM HEPES pH 7.5, 150 mM NaCl over a concentration range of 48–192 μM. A Wyatt Dawn HELEOS-II MALS detector and Wyatt Optilab rEX refractive index monitor recorded both the refractive index and light scattering once separated via SEC. ASTRA software (Wyatt Technology) was used for data analysis.

### HHIP-C mutant heparin affinity chromatography

HHIP-C constructs (200 μg/run) were loaded onto a 1 mL heparin column (HiTrap heparin HP, GE Healthcare) in a buffer of 10 mM HEPES pH 7.5, 40 mM NaCl (~5 mS/cm) and eluted over 30 column volumes with a gradient of elution buffer up to 10 mM HEPES pH 7.5, 1000 mM NaCl (~87 mS/cm). Elution was followed by absorption at 280 nm. The HHIP-C Glu mutant eluted from the column at ~240 mM NaCl, 10 mM HEPES pH 7.5 (22 mS/cm). The dual GAG site mutant (ΔGAG sites 1 + 2) eluted at ~320 mM NaCl, 10 mM HEPES pH 7.5 (29 mS/cm). The β-propeller interface GAG mutant (ΔGAG site 1) eluted at ~400 mM NaCl, 10 mM HEPES pH 7.5 (36 mS/cm) and the EGF interface GAG mutant (ΔGAG site 2) eluted at ~410 mM NaCl, 10 mM HEPES pH 7.5 (37 mS/cm). Wild-type HHIP-C eluted at ~450 mM NaCl, 10 mM HEPES pH 7.5 (40 mS/cm).

### Generation of NIH/3T3 *Extl3*^−/−^ fibroblasts

Clonal EXTL3 knockout cell lines were generated using a double-cut CRISPR strategy. Guides were cloned into pSpCas9(BB)-2A-GFP (PX458; Addgene #48138) and pSpCas9(BB)-2A-mCherry. Plasmids were co-transfected into NIH/3T3 Flp-Ins using X-tremeGENE 9 (Roche). Four days post-transfection, GFP and mCherry double-positive single cells were sorted into 96-well plates using a Sony Cell Sorter Model SH800S. To screen clonal cell lines, we used PCR to detect successful excision of the genomic DNA between the two sgRNA cut sites (Supplementary Fig. 12, Supplementary Table 3).

### HS20 cell staining of CRISPR generated EXTL3 knockouts

To confirm that *Extl3*^−/−^ NIH/3T3 cells had reduced cell surface Heparan Sulfate Proteoglycans (HSPGs), we stained intact cells with a previously characterized HS20 scFv antibody (fused to a 1D4 epitope tag), which recognizes a Heparan Sulphate chains attached to HSPGs^54,55^. Cells were trypsinized and resuspended in Staining Buffer (SB: PBS + 2% BSA + 0.05% sodium azide) at a density of 500,000 cells/100 μL buffer. Cells were then spun down and resuspended in SB with 5% donkey serum. Following a ten-minute room temperature incubation, cells were spun down, resuspended in 50 μL SB containing HS20-1D4 tag (1:20.83 dilution), and incubated for 30 minutes at 4 °C. Samples were then washed twice with SB, before being incubated for 30 min at 4 °C in anti-1D4 monoclonal antibody diluted in SB (1:500; The University of British Columbia). After incubation with anti-1D4 antibody, cells were again washed twice with SB. Samples were then incubated in donkey anti-mouse antibody coupled to Alexa Fluor 594 diluted in SB (1:500; ThermoFisher Scientific A-21203) for 30 minutes at 4 °C. Cells were then washed three times with SB before being resuspended in PBS for flow cytometry analysis. Flow cytometry was performed on a BD Acuri C6 Flow Cytometer using a 552 nm laser for excitation and a 610/20 nm bandpass filter to collect emitted light. Forward and side scatter plots were used to select a population of live, mostly single cells, which were then analyzed. A population of at least 7500 selected cells was analyzed for each sample.

### HH signalling assay

Gli1 mRNA measurements were used as a readout for HH pathway activation and were performed as in^7^. Briefly, NIH/3T3 cells (ATCC, CRL-1658) were grown to confluency and then serum-starved for 24 h via reduction of serum to 0.5% NBCS. The HH pathway was either induced by adding 50 nM purified ShhN_C24II_^12,100^, or NIH/3T3 cells were co-cultured with the full-length HH-producing human cell line PANC-1 (ATCC, CRL-1469)^48^, with addition of HHIP-C constructs or HHIP-∆Hx at 100 nM in each case. For HH pathway stimulation by the palmitoylated N-terminal ShhN peptide (Palm-ShhN15), peptides were added at 10 µM and HHIP-∆Hx at 1 µM (*n* = 2). Reverse transcription quantitative PCR (RT-qPCR) was carried out with the Power SYBR® Green Cells-to-CT^TM^ kit (Life Technologies) according to manufacturer’s instructions. Every experiment was performed with two biological replicates and three technical replicates and results were calculated according to the ΔΔCT method and maximal pathway activation was normalised to 100. The PCR primers for *Gli1* (forward primer, 5′-ccaagccaactttatgtcaggg-3′; reverse primer, 5′-agcccgcttctttgttaatttga-3′); and *Gapdh* (forward primer, 5′-agtggcaaagtggagatt-3′; reverse primer, 5′-gtggagtcatactggaaca-3′) are specific for murine DNAs.

### Reporting summary

Further information on research design is available in the Nature Research Reporting Summary linked to this article.

## Supplementary information

Supplementary Information

Reporting Summary

## Data Availability

Atomic coordinates and structure factors for HHIP-N:SOS, HHIP-N apo, HHIP-C:heparin and HHIP-C:SOS have been deposited in the Protein Data Bank (PDB) (www.rcsb.org) under accession numbers 7PGK, 7PGL, 7PGM and 7PGN, respectively. Any other data supporting the findings of this manuscript are available from the corresponding author upon reasonable request. Source data are provided with this paper.

## Source data

Source Data

## References

[1] Briscoe J, Thérond PP (2013). The mechanisms of Hedgehog signalling and its roles in development and disease. Nat. Rev. Mol. Cell Biol..

[2] Kong, J. H., Siebold, C. & Rohatgi, R. Biochemical mechanisms of vertebrate hedgehog signaling. *Development (Cambridge, England)* **146**, 10.1242/dev.166892 (2019). 10.1242/dev.166892 PMC6550017 31092502

[3] Rubin LL, de Sauvage FJ (2006). Targeting the Hedgehog pathway in cancer. Nat. Rev. Drug Disco..

[4] Wu F, Zhang Y, Sun B, McMahon AP, Wang Y (2017). Hedgehog signaling: from basic biology to cancer therapy. Cell Chem. Biol..

[5] Porter JA (1996). Hedgehog patterning activity: role of a lipophilic modification mediated by the carboxy-terminal autoprocessing domain. Cell.

[6] Pepinsky RB (1998). Identification of a palmitic acid-modified form of human Sonic hedgehog. J. Biol. Chem..

[7] Gong, X. et al. Structural basis for the recognition of Sonic Hedgehog by human Patched1. *Science (New York, N.Y.)* **361**, 10.1126/science.aas8935 (2018). 10.1126/science.aas8935 29954986

[8] Qi X, Schmiege P, Coutavas E, Wang J, Li X (2018). Structures of human Patched and its complex with native palmitoylated sonic hedgehog. Nature.

[9] Qi, X., Schmiege, P., Coutavas, E. & Li, X. Two Patched molecules engage distinct sites on Hedgehog yielding a signaling-competent complex. *Science (New York, N.Y.)* **362**, 10.1126/science.aas8843 (2018). 10.1126/science.aas8843 PMC6341491 30139912

[10] Zhang Y (2018). Structural basis for cholesterol transport-like activity of the Hedgehog receptor patched. Cell.

[11] Qi C, Di Minin G, Vercellino I, Wutz A, Korkhov VM (2019). Structural basis of sterol recognition by human hedgehog receptor PTCH1. Sci. Adv..

[12] Rudolf AF (2019). The morphogen Sonic hedgehog inhibits its receptor Patched by a pincer grasp mechanism. Nat. Chem. Biol..

[13] Luchetti, G. et al. Cholesterol activates the G-protein coupled receptor Smoothened to promote Hedgehog signaling. *eLife* **5**, 10.7554/eLife.20304 (2016). 10.7554/eLife.20304 PMC5123864 27705744

[14] Huang P (2016). Cellular cholesterol directly activates smoothened in Hedgehog signaling. Cell.

[15] Myers BR (2013). Hedgehog pathway modulation by multiple lipid binding sites on the smoothened effector of signal response. Dev. cell.

[16] Bidet M (2011). The hedgehog receptor patched is involved in cholesterol transport. PLoS ONE.

[17] Beachy PA, Hymowitz SG, Lazarus RA, Leahy DJ, Siebold C (2010). Interactions between Hedgehog proteins and their binding partners come into view. Genes Dev..

[18] Rubin JB, Choi Y, Segal RA (2002). Cerebellar proteoglycans regulate sonic hedgehog responses during development. Dev. (Camb., Engl.).

[19] Whalen DM, Malinauskas T, Gilbert RJC, Siebold C (2013). Structural insights into proteoglycan-shaped Hedgehog signaling. Proc. Natl Acad. Sci. USA.

[20] Eaton S (2008). Multiple roles for lipids in the Hedgehog signalling pathway. Nat. Rev. Mol. Cell Biol..

[21] Ohlig S (2011). Sonic hedgehog shedding results in functional activation of the solubilized protein. Dev. Cell.

[22] McLellan JS (2008). The mode of Hedgehog binding to Ihog homologues is not conserved across different phyla. Nature.

[23] Kavran JM, Ward MD, Oladosu OO, Mulepati S, Leahy DJ (2010). All mammalian Hedgehog proteins interact with cell adhesion molecule, down-regulated by oncogenes (CDO) and brother of CDO (BOC) in a conserved manner. J. Biol. Chem..

[24] Allen BL (2011). Overlapping roles and collective requirement for the coreceptors GAS1, CDO, and BOC in SHH pathway function. Dev. Cell.

[25] Izzi L (2011). Boc and Gas1 each form distinct Shh receptor complexes with Ptch1 and are required for Shh-mediated cell proliferation. Developmental cell.

[26] Bishop B (2009). Structural insights into hedgehog ligand sequestration by the human hedgehog-interacting protein HHIP. Nat. Struct. Mol. Biol..

[27] Bosanac I (2009). The structure of SHH in complex with HHIP reveals a recognition role for the Shh pseudo active site in signaling. Nat. Struct. Mol. Biol..

[28] Holtz AM (2015). Secreted HHIP1 interacts with heparan sulfate and regulates Hedgehog ligand localization and function. J. Cell Biol..

[29] Kwong L, Bijlsma MF, Roelink H (2014). Shh-mediated degradation of Hhip allows cell autonomous and non-cell autonomous Shh signalling. Nat. Commun..

[30] Holtz AM (2013). Essential role for ligand-dependent feedback antagonism of vertebrate hedgehog signaling by PTCH1, PTCH2 and HHIP1 during neural patterning. Dev. (Camb., Engl.).

[31] Chuang PT, Kawcak T, McMahon AP (2003). Feedback control of mammalian Hedgehog signaling by the Hedgehog-binding protein, Hip1, modulates Fgf signaling during branching morphogenesis of the lung. Genes Dev..

[32] Chuang PT, McMahon AP (1999). Vertebrate Hedgehog signalling modulated by induction of a Hedgehog-binding protein. Nature.

[33] Wilson NH, Stoeckli ET (2013). Sonic hedgehog regulates its own receptor on postcrossing commissural axons in a glypican1-dependent manner. Neuron.

[34] Olsen CL, Hsu PP, Glienke J, Rubanyi GM, Brooks AR (2004). Hedgehog-interacting protein is highly expressed in endothelial cells but down-regulated during angiogenesis and in several human tumors. BMC Cancer.

[35] Prokopenko D (2018). Whole-genome sequencing in severe chronic obstructive pulmonary disease. Am. J. Respir. Cell Mol. Biol..

[36] Bazan JF, de Sauvage FJ (2009). Structural ties between cholesterol transport and morphogen signaling. Cell.

[37] Aricescu AR, Lu W, Jones EY (2006). A time- and cost-efficient system for high-level protein production in mammalian cells. Acta Crystallogr. Sect. D., Biol. Crystallogr..

[38] El Omari K (2014). Pushing the limits of sulfur SAD phasing: de novo structure solution of the N-terminal domain of the ectodomain of HCV E1. Acta Crystallogr. Sect. D., Biol. Crystallogr..

[39] Hendrickson WA (2014). Anomalous diffraction in crystallographic phase evaluation. Q. Rev. biophysics.

[40] Debreczeni JE, Bunkoczi G, Ma Q, Blaser H, Sheldrick GM (2003). In-house measurement of the sulfur anomalous signal and its use for phasing. Acta Crystallogr. Sect. D..

[41] Chen C (2013). Structural basis for molecular recognition of folic acid by folate receptors. Nature.

[42] Monaco HL (1997). Crystal structure of chicken riboflavin-binding protein. EMBO J..

[43] Byrne, E. F. X. et al. Structural basis of Smoothened regulation by its extracellular domains. *Nature*, 10.1038/nature18934 (2016). 10.1038/nature18934 PMC4970916 27437577

[44] Kwon HJ (2009). Structure of N-terminal domain of NPC1 reveals distinct subdomains for binding and transfer of cholesterol. Cell.

[45] Janda CY, Waghray D, Levin AM, Thomas C, Garcia KC (2012). Structural basis of Wnt recognition by Frizzled. Science (N. Y.).

[46] Nachtergaele S (2013). Structure and function of the Smoothened extracellular domain in vertebrate Hedgehog signaling. eLife.

[47] Han L (2016). Divergent evolution of vitamin B9 binding underlies Juno-mediated adhesion of mammalian gametes. Curr. Biol..

[48] Konitsiotis AD (2014). Attenuation of Hedgehog acyltransferase-catalyzed sonic Hedgehog palmitoylation causes reduced signaling, proliferation and invasiveness of human carcinoma cells. PLoS ONE.

[49] Tukachinsky, H., Petrov, K., Watanabe, M. & Salic, A. Mechanism of inhibition of the tumor suppressor Patched by Sonic Hedgehog. *Proc. Natl Acad. Sci. USA*, 201606719, 10.1073/pnas.1606719113 (2016). 10.1073/pnas.1606719113 PMC5056095 27647915

[50] Luque I, Freire E (1998). Structure-based prediction of binding affinities and molecular design of peptide ligands. Methods Enzymol..

[51] Mulloy B, Forster MJ (2000). Conformation and dynamics of heparin and heparan sulfate. Glycobiology.

[52] Waksman G, Herr AB (1998). New insights into heparin-induced FGF oligomerization. Nat. Struct. Biol..

[53] Lebensohn, A. M. & Rohatgi, R. R-spondins can potentiate WNT signaling without LGRs. *eLife* **7**, 10.7554/eLife.33126 (2018). 10.7554/eLife.33126 PMC5800842 29405118

[54] Dubey, R. et al. R-spondins engage heparan sulfate proteoglycans to potentiate WNT signaling. *eLife* **9**, 10.7554/eLife.54469 (2020). 10.7554/eLife.54469 PMC7239654 32432544

[55] Gao W (2014). Inactivation of Wnt signaling by a human antibody that recognizes the heparan sulfate chains of glypican-3 for liver cancer therapy. Hepatology.

[56] Janda CY, Garcia KC (2015). Wnt acylation and its functional implication in Wnt signalling regulation. Biochem Soc. Trans..

[57] Kowatsch C, Woolley RE, Kinnebrew M, Rohatgi R, Siebold C (2019). Structures of vertebrate Patched and Smoothened reveal intimate links between cholesterol and Hedgehog signalling. Curr. Opin. Struct. Biol..

[58] Qi X, Li X (2020). Mechanistic insights into the generation and transduction of Hedgehog signaling. Trends Biochem Sci..

[59] Coulombe J, Traiffort E, Loulier K, Faure H, Ruat M (2004). Hedgehog interacting protein in the mature brain: membrane-associated and soluble forms. Mol. Cell. Neurosci..

[60] Lin X (2004). Functions of heparan sulfate proteoglycans in cell signaling during development. Dev. (Camb., Engl.).

[61] Maun HR (2010). Hedgehog pathway antagonist 5E1 binds hedgehog at the pseudo-active site. J. Biol. Chem..

[62] Bailey JM (2008). Sonic hedgehog promotes desmoplasia in pancreatic cancer. Clin. Cancer Res.

[63] Xu D, Esko JD (2014). Demystifying heparan sulfate-protein interactions. Annu Rev. Biochem.

[64] Chang VT (2007). Glycoprotein structural genomics: solving the glycosylation problem. Struct. (Lond., Engl.: 1993).

[65] Walter TS (2005). A procedure for setting up high-throughput nanolitre crystallization experiments. Crystallization workflow for initial screening, automated storage, imaging and optimization. Acta Crystallogr. Sect. D. Biol. Crystallogr..

[66] Liu ZJ (2011). A multi-dataset data-collection strategy produces better diffraction data. Acta Crystallogr. Sect. A, Found. Crystallogr..

[67] Evans P (2006). Scaling and assessment of data quality. Acta Crystallogr. Sect. D. Biol. Crystallogr..

[68] Kabsch W (1988). Automatic indexing of rotation diffraction patterns. J. Appl. Crystallogr..

[69] Leslie AG (2006). The integration of macromolecular diffraction data. Acta Crystallogr. Sect. D., Biol. Crystallogr..

[70] Sauter NK, Grosse-Kunstleve RW, Adams PD (2004). Robust indexing for automatic data collection. J. Appl. Crystallogr..

[71] Winter G (2010). xia2: an expert system for macromolecular crystallography data reduction. J. Appl. Crystallogr..

[72] Zhang Z, Sauter NK, van den Bedem H, Snell G, Deacon AM (2006). Automated diffraction image analysis and spot searching for high-throughput crystal screening. J. Appl. Crystallogr..

[73] Gildea RJ (2014). New methods for indexing multi-lattice diffraction data. Acta Crystallogr. Sect. D. Biol. Crystallogr..

[74] Karplus PA, Diederichs K (2012). Linking crystallographic model and data quality. Science (N. Y.).

[75] Pape T, Schneider TR (2004). HKL2MAP: a graphical user interface for macromolecular phasing with SHELX programs. J. Appl. Crystallogr..

[76] Sheldrick GM (2010). Experimental phasing with SHELXC/D/E: combining chain tracing with density modification. Acta Crystallogr. Sect. D. Biol. Crystallogr..

[77] Terwilliger TC (2009). Decision-making in structure solution using Bayesian estimates of map quality: the PHENIX AutoSol wizard. Acta Crystallogr. Sect. D. Biol. Crystallogr..

[78] Terwilliger TC (2008). Iterative model building, structure refinement and density modification with the PHENIX AutoBuild wizard. Acta Crystallogr. Sect. D. Biol. Crystallogr..

[79] Read RJ, McCoy AJ (2011). Using SAD data in Phaser. Acta Crystallogr. Sect. D. Biol. Crystallogr..

[80] Emsley P, Cowtan K (2004). Coot: model-building tools for molecular graphics. Acta Crystallogr. Sect. D. Biol. Crystallogr..

[81] Murshudov GN (2011). REFMAC5 for the refinement of macromolecular crystal structures. Acta Crystallogr. Sect. D..

[82] Smart OS (2012). Exploiting structure similarity in refinement: automated NCS and target-structure restraints in BUSTER. Acta Crystallogr. Sect. D. Biol. Crystallogr..

[83] Adams PD (2010). PHENIX: a comprehensive Python-based system for macromolecular structure solution. Acta Crystallogr. Sect. D. Biol. Crystallogr..

[84] McCoy AJ (2007). Phaser crystallographic software. J. Appl Crystallogr.

[85] Agirre J (2015). Privateer: software for the conformational validation of carbohydrate structures. Nat. Struct. Mol. Biol..

[86] Davis IW (2007). MolProbity: all-atom contacts and structure validation for proteins and nucleic acids. Nucleic acids Res..

[87] Baker NA, Sept D, Joseph S, Holst MJ, McCammon JA (2001). Electrostatics of nanosystems: application to microtubules and the ribosome. Proc. Natl Acad. Sci. USA.

[88] Krissinel E, Henrick K (2007). Inference of macromolecular assemblies from crystalline state. J. Mol. Biol..

[89] Laskowski RA (2001). PDBsum: summaries and analyses of PDB structures. Nucleic Acids Res..

[90] Riffel N (2002). Atomic resolution structure of moloney murine leukemia virus matrix protein and its relationship to other retroviral matrix proteins. Struct. (Lond., Engl.: 1993).

[91] Stuart DI, Levine M, Muirhead H, Stammers DK (1979). Crystal structure of cat muscle pyruvate kinase at a resolution of 2.6 A. J. Mol. Biol..

[92] Felsenstein, J. PHYLIP: Phylogeny Inference Package. Version 3.2. Joel Felsenstein. **64**, 539–541, (1989).

[93] Pettersen EF (2004). UCSF Chimera–a visualization system for exploratory research and analysis. J. Comput. Chem..

[94] Bond CS, Schuttelkopf AW (2009). ALINE: a WYSIWYG protein-sequence alignment editor for publication-quality alignments. Acta Crystallogr. Sect. D., Biol. Crystallogr..

[95] Brown PH, Schuck P (2006). Macromolecular size-and-shape distributions by sedimentation velocity analytical ultracentrifugation. Biophys. J..

[96] Malinauskas T, Aricescu AR, Lu W, Siebold C, Jones EY (2011). Modular mechanism of Wnt signaling inhibition by Wnt inhibitory factor 1. Nat. Struct. Mol. Biol..

[97] O’Callaghan C A (1999). BirA enzyme: production and application in the study of membrane receptor-ligand interactions by site-specific biotinylation. Anal. Biochem..

[98] Chazotte, B. Labeling nuclear DNA with hoechst 33342. *Cold Spring Harbor protocols* **2011**, pdb.prot5557 (2011). 10.1101/pdb.prot5557 21205857

[99] Schindelin J (2012). Fiji: an open-source platform for biological-image analysis. Nat. Methods.

[100] Taylor FR (2001). Enhanced potency of human sonic hedgehog by hydrophobic modification. Biochemistry.

