# Supplementary material for: Hedgehog-Interacting Protein is a multimodal antagonist of Hedgehog signalling
Source: Nat Commun. 2021 Dec 9;12:7171. doi: 10.1038/s41467-021-27475-2 (PMC8660895; doi:10.1038/s41467-021-27475-2)
Supplement: Supplementary file 1 — Supplementary Information [file 41467_2021_27475_MOESM1_ESM.pdf]

Supplementary Information for '**Hedgehog-Interacting Protein is a multimodal antagonist of Hedgehog signalling**'

Samuel C. Griffiths[1,5#], Rebekka A. Schwab[1,5], Kamel El Omari[2], Benjamin Bishop[1], Ellen J. Iverson[3], Tomas Malinauskas[1], Ramin Dubey[3], Mingxing Qian[4], Douglas F. Covey[4], Robert J.C. Gilbert[1], Rajat Rohatgi[3] & Christian Siebold[1*]

[1]Division of Structural Biology, Wellcome Centre for Human Genetics, University of Oxford, OX3 7BN, Oxford UK.

[2]Science Division, Diamond Light Source, Harwell Science and Innovation Campus, Didcot, OX11 0DE, UK.

[3]Departments of Biochemistry and Medicine, Stanford University School of Medicine, Stanford, California, United States of America.

[4]Department of Developmental Biology, Washington University School of Medicine, St. Louis, Missouri, United States of America.

[5]These authors contributed equally: Samuel C. Griffiths, Rebekka A. Schwab.

[#]Current address: Evotec (UK) Ltd., Milton Park, Abingdon, OX14 4RZ, UK.

[*]Corresponding author: christian@strubi.ox.ac.uk (ORCID: 0000-0002-6635-3621).

Supplementary Table 1. Data collection and refinement statistics for HHIP structures.

	HHIP-N:SOS	HHIP-N:apo	HHIP-N:SOS (S-SAD)	HHIP-C: heparin	HHIP-C:SOS
**Data collection**					
Space group	$P4_22_12$	$P4_32_12$	$P4_22_12$	C2	$P3_121$
Cell dimensions					
a, b, c (Å)	60.4, 60.4, 108.5	59.1, 59.1, 98.7	59.8, 59.8,108.7	170.6, 105.3, 148.4	100.6, 100.6, 311.9
α, β, γ (°)	90.0, 90.0, 90.0	90.0, 90.0, 90.0	90.0, 90.0, 90.0	90.0, 96.0, 90.0	90.0, 90.0, 120.0
No. crystals	1	1	8	1	1
Wavelength (Å)	0.97620	0.96860	1.77120	0.97950	0.97949
Resolution (Å)	54.20-2.75 (2.85-2.75)	50.67-2.63 (2.70-2.63)	108.70-2.90 (3.00-2.90)	147.60-2.70 (2.75-2.70)	76.00-2.40 (2.44-2.40)
No. unique reflections	5609 (534)	5640 (420)	4801 (345)	71834 (3487)	72635 (3547)
Completeness (%)	100.0 (100.0)	100.0 (100.0)	100.0 (100.0)	99.9 (98.4)	100.0 (99.9)
Multiplicity	11.9 (10.9)	12.3 (12.3)	222.4 (20.9)	6.6 (5.8)	19.9 (20.0)
$\langle I/\sigma(\mathrm{I})\rangle$	12.0 (1.1)	22.3 (1.4)	41.7 (2.0)	12.6 (0.8)	21.4 (0.8)
R_{merge} (%)	11.3 (182.7)	5.8 (180.7)	19.8 (137.3)	7.7 (221.8)	9.7 (431.9)
R_{pim} (%)	3.5 (57.4)	2.4 (75.2)	1.2 (30.9)	3.3 (100.5)	2.2 (97.7)
$CC_{1/2}$	1.0 (0.4)	1.0 (0.5)	1.0 (0.8)	1.0 (0.3)	1.0 (0.3)
**Refinement**					
Resolution	54.0-2.75	50.67-2.63	-	84.8–2.7	58.0-2.4
No. reflections (test set)	5609 (288)	5602 (281)	-	70890 (3635)	72499 (3664)
R_{work} / R_{free} (%)	26.7 / 28.6	24.4 / 26.5	-	21.6 / 24.0	19.0 / 22.7
No. atoms:					
Protein	964	807	-	10335	6843
SOS	55	-	-	-	220
Heparin	-	-	-	140	
Water	-	-	-	-	37
Ca^{2+}	-	-	-	-	2
Glycerol	-	-	-		6
Average B factor (Å^2):					
Protein	97.0	106.4	-	96.5	95.3
SOS	195.2		-	-	239.8
Heparin			-	193.1	-
Water			-	-	70.8
Ca^{2+}			-	-	136.6
Glycerol			-	-	108.8
RMSD bond lengths (Å)	0.007	0.002	-	0.004	0.008
RMSD bond angles (°)	0.9	0.5	-	0.9	1.0
Ramachandran plot (%)					
Favoured	97.3	97.9	-	98.2	98.7
Allowed	2.7	2.1	-	1.72	1.3
Outliers	0	0	-	0.08	0

Values in parentheses are for highest-resolution shell. RMSD: Root Mean Square Deviation.

Supplementary Table 2. Evolutionary analysis of CRD structures.

Protein	Fz8-PAM (4F0A)[A]	Smo (5L7D)	Fz8 (1IJY)	sFRP3 (1IJX)	MuSK (3HKL)	NPC1 (3GKI)	RFBP (not in PDB)	FRα (4LRH)	JUNO (5EJN)	FRβ (4KMZ)
HHIP-N CRD	2.20[B]<br>38[C]<br>14.94[D]	2.35<br>41<br>13.57	2.27<br>42<br>14.35	2.24<br>41<br>14.36	1.84<br>51<br>23.13	1.99<br>61<br>19.7	1.53<br>71<br>34.69	1.53<br>66<br>34.33	1.54<br>63<br>32.47	1.55<br>66<br>34.83
Fz8-PAM (4F0A)		1.00<br>83<br>43.64	0.16<br>117<br>102.54	0.40<br>106<br>79.01	1.30<br>78<br>38.33	2.17<br>61<br>21.36	2.54<br>40<br>13.35	2.76<br>44<br>12.16	2.51<br>44<br>13.31	2.43<br>43<br>14.14
Smo (5L7D)			1.09<br>84<br>43.18	1.04<br>80<br>44.60	1.26<br>78<br>40.84	2.32<br>54<br>19.01	2.68<br>46<br>11.24	2.62<br>46<br>13.73	2.64<br>44<br>12.47	2.49<br>48<br>15.65
Fz8 (1IJY)				0.45<br>108<br>78.07	1.35<br>76<br>36.13	2.14<br>62<br>22.27	2.50<br>45<br>15.00	2.66<br>43<br>13.41	2.38<br>45<br>15.40	2.53<br>43<br>14.34
sFRP3 (1IJX)					1.36<br>79<br>36.65	2.41<br>60<br>17.28	2.31<br>45<br>17.60	2.59<br>47<br>14.13	2.49<br>39<br>13.87	2.47<br>46<br>15.62
MuSK (3HKL)						2.14<br>67<br>22.48	2.25<br>55<br>20.03	2.30<br>55<br>15.14	2.16<br>53<br>19.21	2.13<br>53<br>23.24
NPC1 (3GKI)							1.73<br>82<br>35.46	1.95<br>89<br>31.57	1.74<br>81<br>36.37	1.74<br>90<br>30.58
RFBP (not in PDB)								0.68<br>160<br>104.06	0.85<br>138<br>80.30	0.66<br>157<br>105.39
FRα (4LRH)									0.42<br>161<br>125.21	0.16<br>194<br>176.05
JUNO (5EJN)										0.46<br>157<br>120.83

[A]PDB accession codes are shown in parenthesis; [B]Root-mean Square Deviation (RMSD) values were calculated for equivalent Cα atom positions using the program SHP [1,2]; [C]Number of equivalent Cα positions utilised in RMSD calculation via SHP; [D]Summed structural correlation (total probability) values calculated using SHP. The phylogenetic tree for analysed CRDs (Fig. 2a) was arranged using the program PHYLIP [3] with these summed structural correlation values to construct a distance matrix. Fz8-PAM – Frizzled 8-palmitoleate complex [4], Smo – Smoothened [5], Fz8 – Frizzled 8 [6], sFRP3 – secreted Frizzled-related protein [6], MuSK – muscle-specific kinase [7], NPC1 – Niemann-Pick C1 protein [8], RFBP – riboflavin-binding protein [9], FRα – folate receptor α [10], FRβ – folate receptor β [11], JUNO – folate receptor δ [12].

Supplementary Table 3. List of primers used in this study.

Primer name	Primer sequence	Background
HHIP-ΔHx_Fwd	CGGCTAG ACCGGT TGCCTGAATGGGAAT CCC	Forward primer to clone HHIP-ΔHx
HHIP-ΔHx_Rev	CGGCTAG GGTACC GTCCACTTGTTCACA TTG	Reverse primer to clone HHIP-ΔHx
HHIP-ΔHx_R185A/K186A/R189A_Fwd	CCAGATTTTCCA GCA GCA CAAGTC GCA GGACCAGCATCT	Forward primer to introduce R185A/K186A/R189A mutations to HHIP-ΔHx via overlap extension PCR
HHIP-ΔHx_R185A/K186A/R189A_Rev	AGATGCTGGTCCTGC GACTTGTGCTGCTGG AAAATCTGG	Reverse primer to introduce R185A/K186A/R189Amutations to HHIP-ΔHx via overlap extension PCR
HHIP-ΔHx_K204A/R210A/K211A/K213A_Fwd	GAATATGAC GCA GTGGAAGAGATCAG C GCA GCG CAC GCA CACAACTGC	Forward primer to introduce K204A/R210A/K211A/K213A mutations to HHIP-ΔHx via overlap extension PCR
HHIP-ΔHx_K204A/R210A/K211A/K213A_Rev	GCAGTTGTGTGCGTG CGCTGCGCTGATCTC TTCCACTGCGTCATA TTC	Forward primer to introduce K204A/R210A/K211A/K213A mutations to HHIP-ΔHx via overlap extension PCR
N-HA-HHIP-ΔHx_Fwd	CGC ACCGGT TACCCATACGATGTT CCAGATTACGCT TGCCTGAATGGGAAT CCC	Forward primer to generate N-HA-tagged HHIP-ΔHx
N-HA-HHIP-C_Fwd	CGC ACCGGT TACCCATACGATGTT CCAGATTACGCT AAACACAACTGCTTC TGTATTC	Forward primer to generate N-HA-tagged HHIP-C
N-HA-HHIP-ΔHx_Rev	CGC GGTACC TCATTAGTCCACTTG TTCACATTG	Reverse primer to generate N-HA-tagged HHIP-ΔHx and HHIP-C (no C-tag)
N-HA-HHIP-N_Fwd	CGC ACCGGT TACCCATACGATGTT CCAGATTACGCT TGCCTGAATGGGAAT CCC	Forward primer to generate N-HA-tagged HHIP-N
N-HA-HHIP-N_Rev	CGC GGTACC TCATTA GCTGATCTCTTCCAC	Reverse primer to generate N-HA-tagged HHIP-N (no C-tag)
HHIP-C_Fwd	CGGCTAG ACCGGT AAACACAACTGCTTC TGTATTC	Forward primer to generate HHIP-C (used in conjunction with HHIP-ΔHx_Rev)
HHIP-C_K277N/G279T_Fwd	CAAAGTGGAATA AAC GGA ACA GATGAAAGAGGACT G	Forward primer (1) to produce ΔGAG site 1 mutant in HHIP-C via overlap extension PCR

HHIP-C_K277N/G279T_Rev	CAGTCCTCTTTCATC TGTTCCGTTTATTCC ACTTTG	Reverse primer (1) to produce ΔGAG site 1 mutant in HHIP-C via overlap extension PCR
HHIP-C_R350N/H352_Fwd	GCAGAACTCCAC AAT AAG ACT CTGGGAGGA	Forward primer (2) to produce ΔGAG site 1 mutant in HHIP-C via overlap extension PCR
HHIP-C_R350N/H352_Rev	TCCTCCCAGAGTCTT ATTGTGGAGTTCTGC	Reverse primer (2) to produce ΔGAG site 1 mutant in HHIP-C via overlap extension PCR
HHIP-C_T603N_Fwd	CAACCTGCACAG AAC CTGACTTCAGAG	Forward primer (1) to produce ΔGAG site 2 mutant in HHIP-C via overlap extension PCR
HHIP-C_T603N_Rev	CTCTGAAGTCAGGTT CTGTGCAGGTTG	Reverse primer (1) to produce ΔGAG site 2 mutant in HHIP-C via overlap extension PCR
HHIP-C_Y616T_Fwd	TGTCGAAACGGC ACC TGCACCCCCACG	Forward primer (2) to produce ΔGAG site 2 mutant in HHIP-C via overlap extension PCR
HHIP-C_Y616T_Rev	CGTGGGGGTGCAGG TGCCGTTTCGACA	Reverse primer (2) to produce ΔGAG site 2 mutant in HHIP-C via overlap extension PCR
HHIP-C_ΔGAG-fusion_Fwd	GATAGACATCCCACT GATATAAAC	Forward primer used to combine ΔGAG site 1 and ΔGAG site 2 mutants into a single chain via overlap extension PCR
HHIP-C_ΔGAG-fusion_Rev	GTTTATATCAGTGGG ATGTCTATC	Reverse primer used to combine ΔGAG site 1 and ΔGAG site 2 mutants into a single chain via overlap extension PCR
CRISPR_guide_Fwd	ACTGTCACAGACTGT TACCG	Forward primer for CRISPR *EXTL3*$^{-/-}$ cell line generation
CRISPR_guide_Rev	TGTGTGCCAATTGTT TGGAG	Reverse primer for CRISPR *EXTL3*$^{-/-}$ cell line generation
CRISPR_seq_Fwd	CGTCACAGAGGTCC ACTTCC	Forward primer for sequencing of *EXTL3*$^{-/-}$ cell line
CRISPR_seq_Rev	GAAAGCCAATGCTG CTCCAC	Reverse primer for sequencing of *EXTL3*$^{-/-}$ cell line

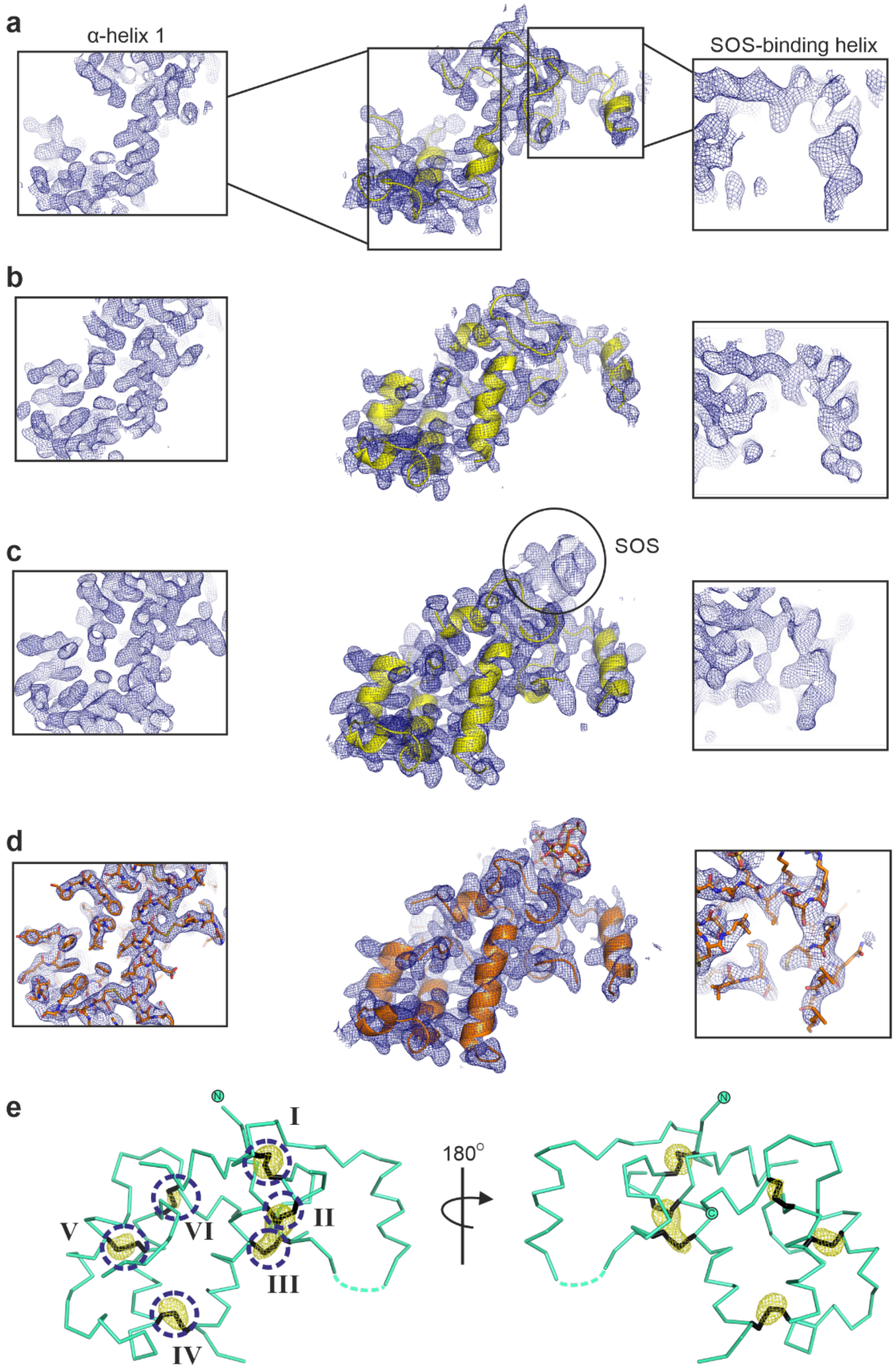

Supplementary Fig. 1. HHIP-N structure solution pipeline. **(a-d)** Models for the major steps of the structure solution process are displayed with the associated $2F_o$-F_c electron density maps contoured at 1σ and displayed as a blue mesh**.** Two well-resolved features of HHIP-N, α1 of the CRD region (α-helix 1, left) and the SOS-binding helix (right), are displayed in boxes. **(a)** Output from the first Phenix Autosol/Autobuild cycle. **(b)** Second Phenix Autosol/Autobuild cycle. **(c)** Second refinement run from *Refmac5* with the first appearance of electron density for a SOS molecule. **(d)** Final refinement step using *AutoBUSTER*. Both C and D were extended to 2.75 Å resolution and the final refined model is coloured orange versus yellow for partially-built models. **(e)** Anomalous difference map calculated from S-SAD data used for phasing is displayed as a yellow mesh contoured at 4σ and mapped onto the refined HHIP-N structure. Disulphide bonds are numbered using Roman numerals.

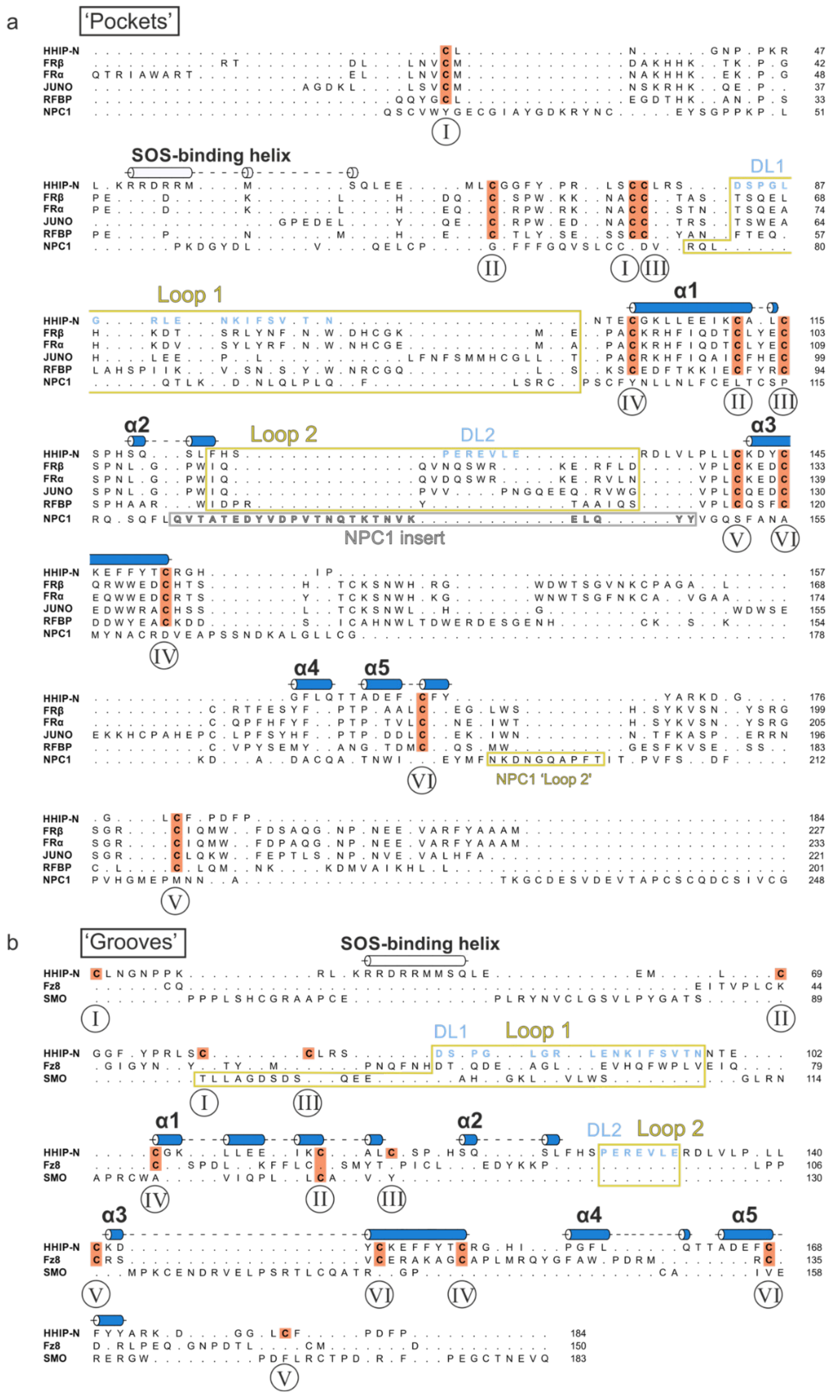

Supplementary Fig. 2. Structure-based sequence alignment of CRDs analysed in Figure 2. **(a)** Pocket-type CRDs. **(b)** Groove-type CRDs. HHIP-N helices are shown above the sequence using cylinders and dashed lines and are annotated as in Fig. 1c, with the 5 helices from the CRD region displayed in blue and the SOS-binding helix from the N-terminal GAG-binding domain in white. Loops 1 and 2 are outlined with yellow boxes, with DL1 and DL2 from HHIP-N highlighted in blue. Disulphide bonds present in HHIP-N are indicated using Roman numerals below sequence and indicated using orange boxes. The NPC1 β-sheet insert (see Fig. 2c) is highlighted in grey and indicated using a grey box. Residues 61-66 are absent in our HHIP-N structure and were not included. Residues from HHIP-N DL1 and DL2 disordered in the structure were added manually.

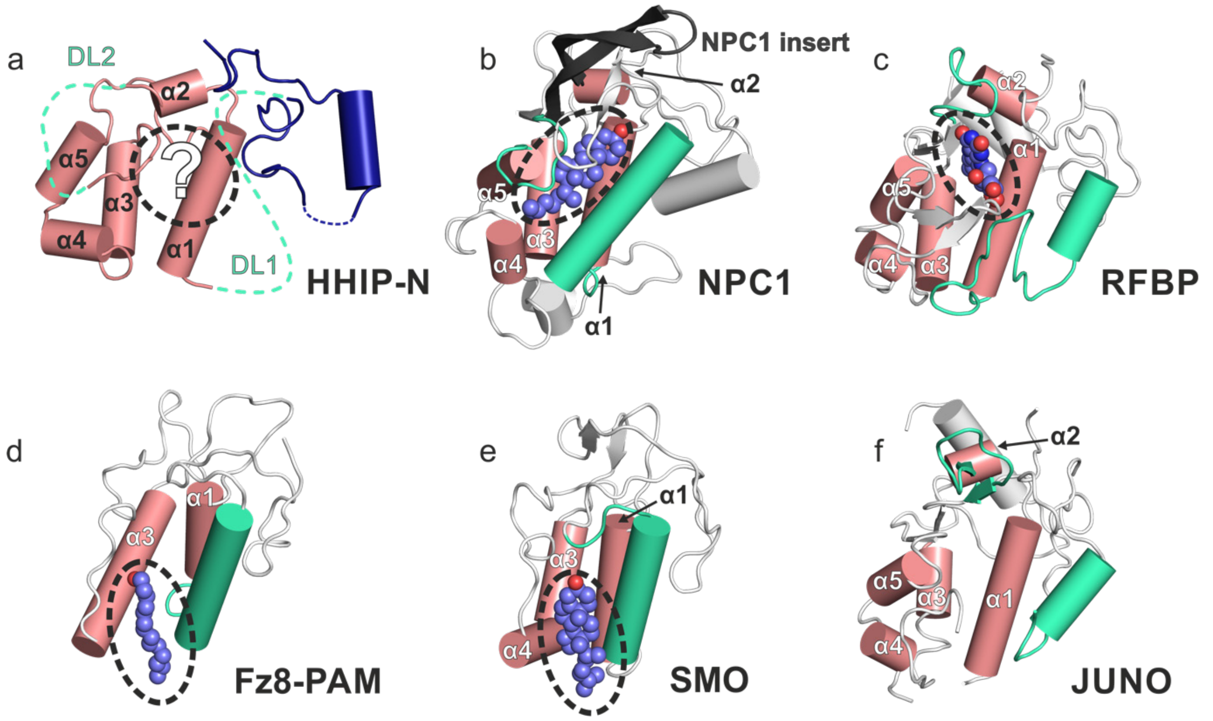

Supplementary Fig. 3. CRD structure comparison. **(a)** HHIP-N coloured as in Fig. 1b, with helices numbered α1-5 (coloured salmon) and DL1 and DL2 shown using green dashed lines. A potential central ligand-binding pocket is displayed using a dashed circle. **(b-f)** Related CRDs (labelled) in the same orientation as (a). Ligand-binding loops are shown in green, and CRD helices superposed with those of HHIP-N are labelled and coloured salmon. Bound ligands (NPC1: cholesterol; SMO: cholesterol; RFBP: riboflavin; Fz8: palmitoleate) are displayed as spheres outlined with a dashed oval.

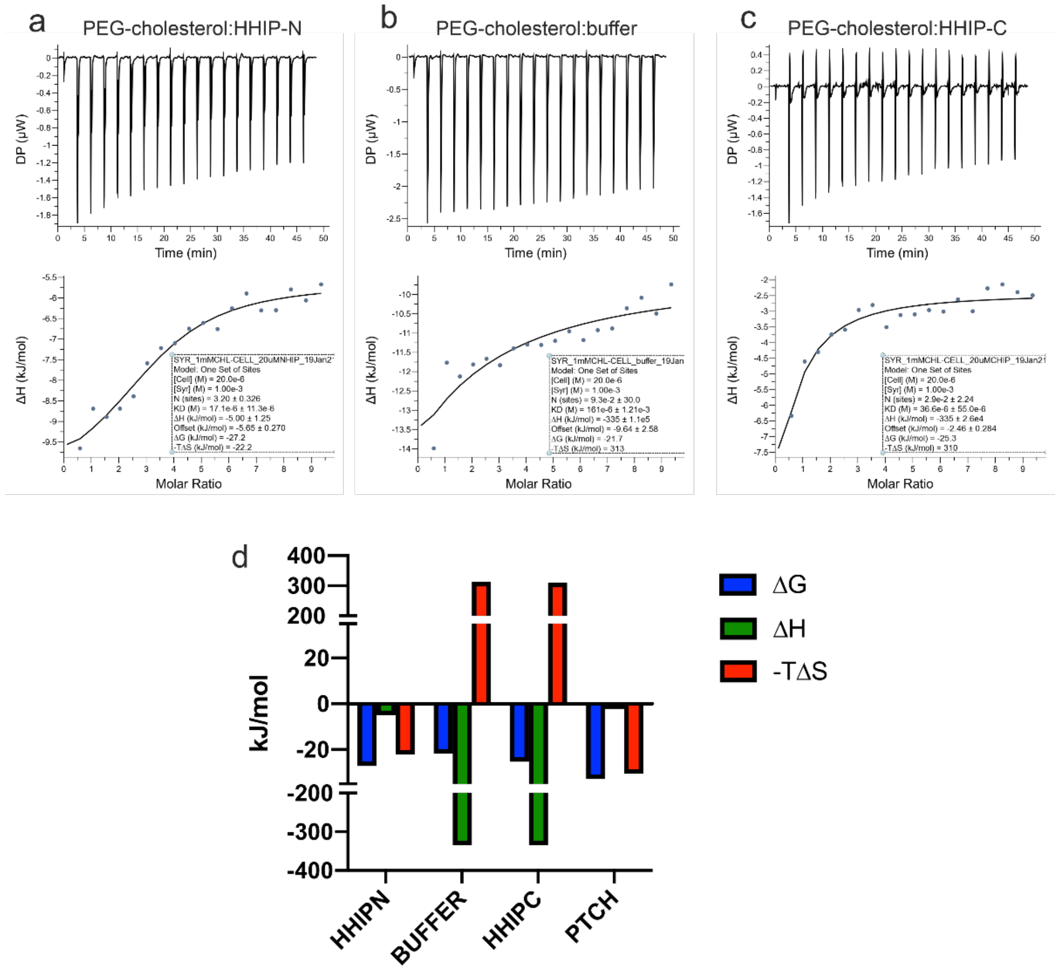

Supplementary Fig. 4. Detailed analysis of the HHIPN:PEG-cholesterol interaction measured by ITC. Raw isotherms obtained for HHIP-N:PEG-cholesterol (**a**, upper panel), buffer:PEG-cholesterol (**b**, upper panel) and HHIP-C:PEG-cholesterol (**c**, upper panel) with corresponding integrated heats of injection (lower panels). HHIP-C was used as a control protein that did not show a specific interaction with PEG-cholesterol. **d,** Thermodynamic signature plots showing Gibbs free energy (ΔG), enthalpy (ΔH) and entropy (-TΔS) for the data shown in a-c with the addition of data from previously published PTCH:PEG-cholesterol data[13]. When the thermodynamic signature of each experiment is examined, the HHIP-N:PEG-cholesterol data reveal that the majority of the binding is due to a favourable enthalpic contribution, corresponding to a mainly hydrophobic interaction with a -TΔS of -22.2 kJ/mol (contributing ~82% of the Gibbs free energy (ΔG)) as is seen for other hydrophobic binding events (see for example[14]). In contrast, the thermodynamic signature of buffer:PEG-cholesterol shows an extremely unfavourable entropic interaction and huge errors for calculated enthalpies concomitant with high background of heat of dilution. As an additional control, PEG-cholesterol was titrated into HHIP-C, which has been shown to bind the globular part of SHH and not the lipid moieties[15,16]. A similar behaviour to that seen for buffer only was observed, with a poor fit and large errors: Kd = 37 μM ±55 μM, ΔH = -335 kJ/mol ±2600 and -TΔS = 310 kJ/mol. The striking difference between the buffer:PEG-cholesterol and HHIP-C:PEG-cholesterol interaction on one side, and the HHIP-N:PEG-cholesterol interaction on the other side is also well-represented by the thermodynamic signature plots for each of the experiments. Here, the HHIP-N:PEG-cholesterol interaction gives reasonable binding statistics with small errors. Importantly, this agrees with previously carried out ITC experiments of Hh receptor PTCH with the same PEG-cholesterol molecule[13].

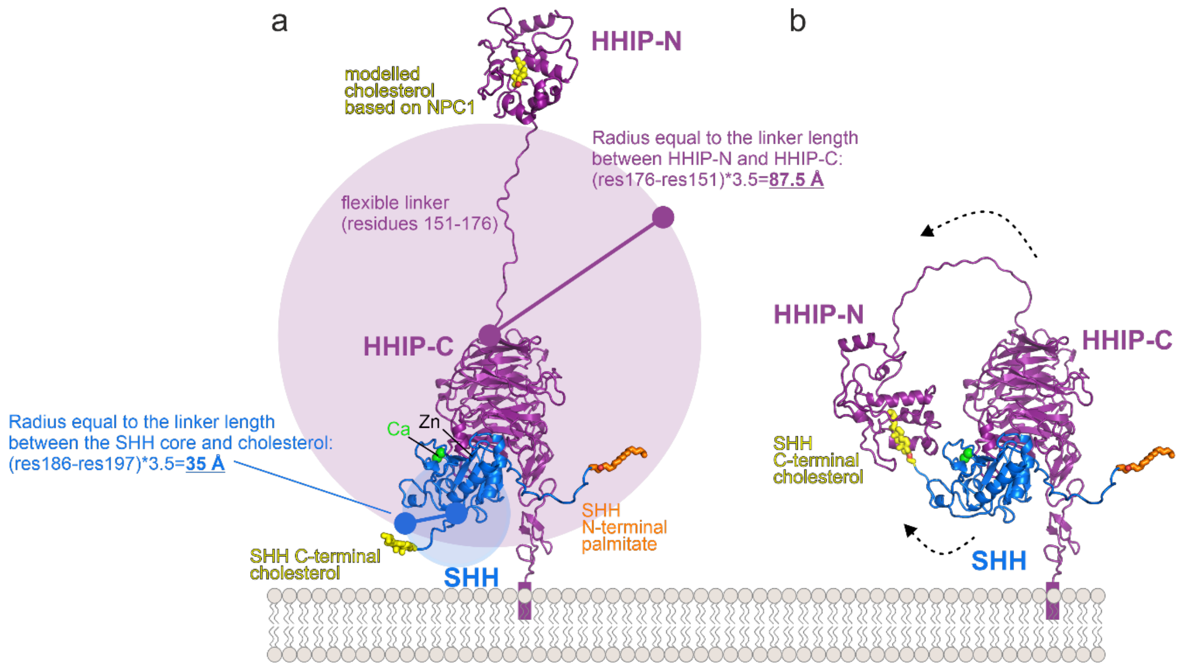

Supplementary Fig. 5. Model of the full-length 1:1 HHIP-SHH complex in an extended (a) and closed (b) conformation. The length of the flexible linker between HHIP-N and HHIP-C (87.5 Å), and the SHH core domain and the cholesterol attachment were calculated estimated by multiplying the number of amino acids by ~3.4-4.0 Å (see following link for details: https://bionumbers.hms.harvard.edu/bionumber.aspx?s=n&v=5&id=114332). The overlap between two circles/spheres suggests that the SHH-attached cholesterol moiety can potentially access the HHIP CRD, thereby forming a 1:1 SHH-HHIP complex.

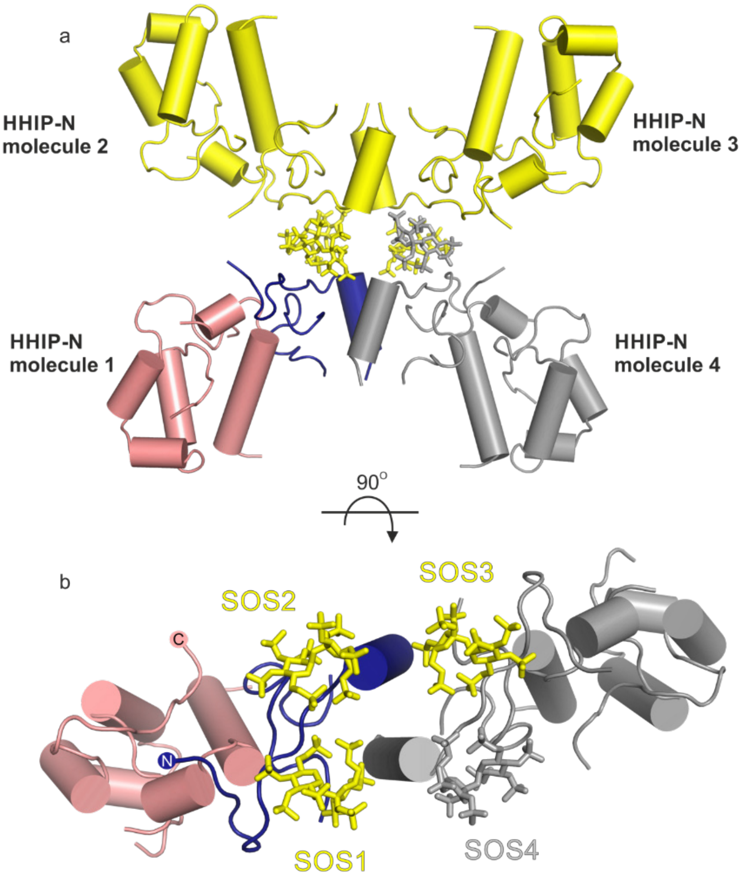

Supplementary Fig. 6. HHIP-N crystal packing analysis. **(a)** Perpendicular view along the 4-fold crystallographic axis. Note that the asymmetric unit contains one HHIP-N and one SOS molecule. **(b)** Coordination of SOS molecules. Each HHIP-N molecule interacts with 3 SOS molecules, e.g. the HHIP-N chain displayed in blue and salmon binds SOS molecules 1, 2 and 3, whereas the grey HHIP-N molecule makes contacts with SOS molecules 1, 3 and 4.

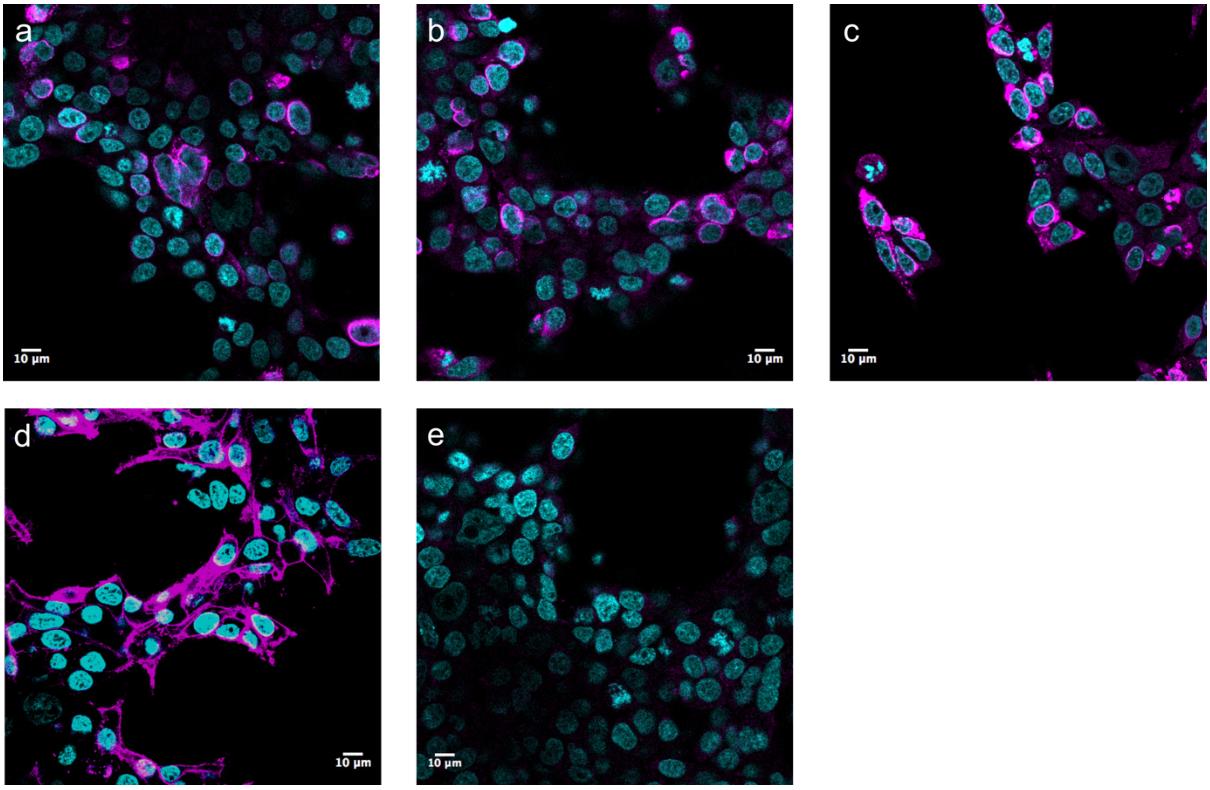

Supplementary Fig. 7. Immunofluorescence microscopy in HEK293T cells of HA-tagged HHIP constructs bound to the cell surface. **(a)** N-HA-HHIP-N; **(b)** N-HA-HHIP-C; **(c)** N-HA-HHIP-ΔHx; **(d)** GPI-anchored N-HA-RGMB [17] and **(e)** secreted GAG-binding Neurotrophin-3 (1D4-tagged) [18] Cells were transfected and fixed before staining with a primary mouse anti-HA antibody (Thermo Fisher) followed by a secondary Alexa Fluor® 633-conjugated anti-Mouse IgG antibody (Thermo Fisher). Hoechst stain was added to stain cell nuclei [19]. Anti-HA antibody staining is displayed in magenta, nuclear staining in cyan. Images shown correspond to representative populations of cells from the dishes imaged.

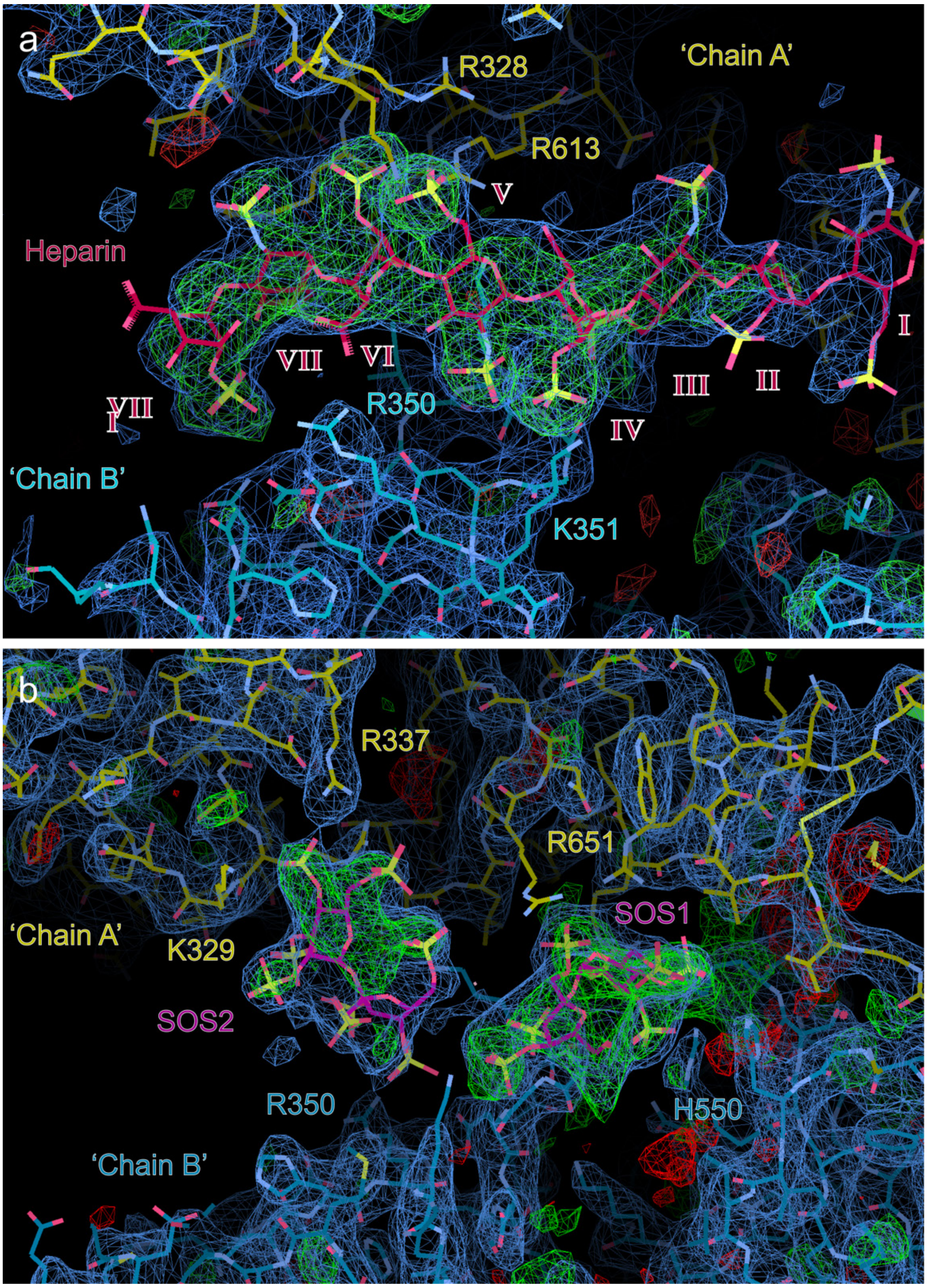

Supplementary Fig. 8. Structure solution of HHIP-C:GAG complexes. View of the 8-mer heparin molecule visible (red, **a**) and SOS molecules (pink, **b**), bound between HHIP-C chain A (EGF repeats; yellow) and a symmetry-related chain B (β-propeller; blue-green). Heparin residues are numbered using Roman numerals (odd, O2-Sulphoiduronic acid; even, N,O6-disulphoglucosamine) and the two SOS molecules are numbered as per Supplementary Fig. 7c (see below). The sigma-A weighted $2F_o$-F_c (1.0 σ, blue) and F_o-F_c (green: +3.0 σ, red: -3.0 σ) electron density maps are displayed. Residues observed to coordinate GAGs are highlighted (see Supplementary Fig. 8).

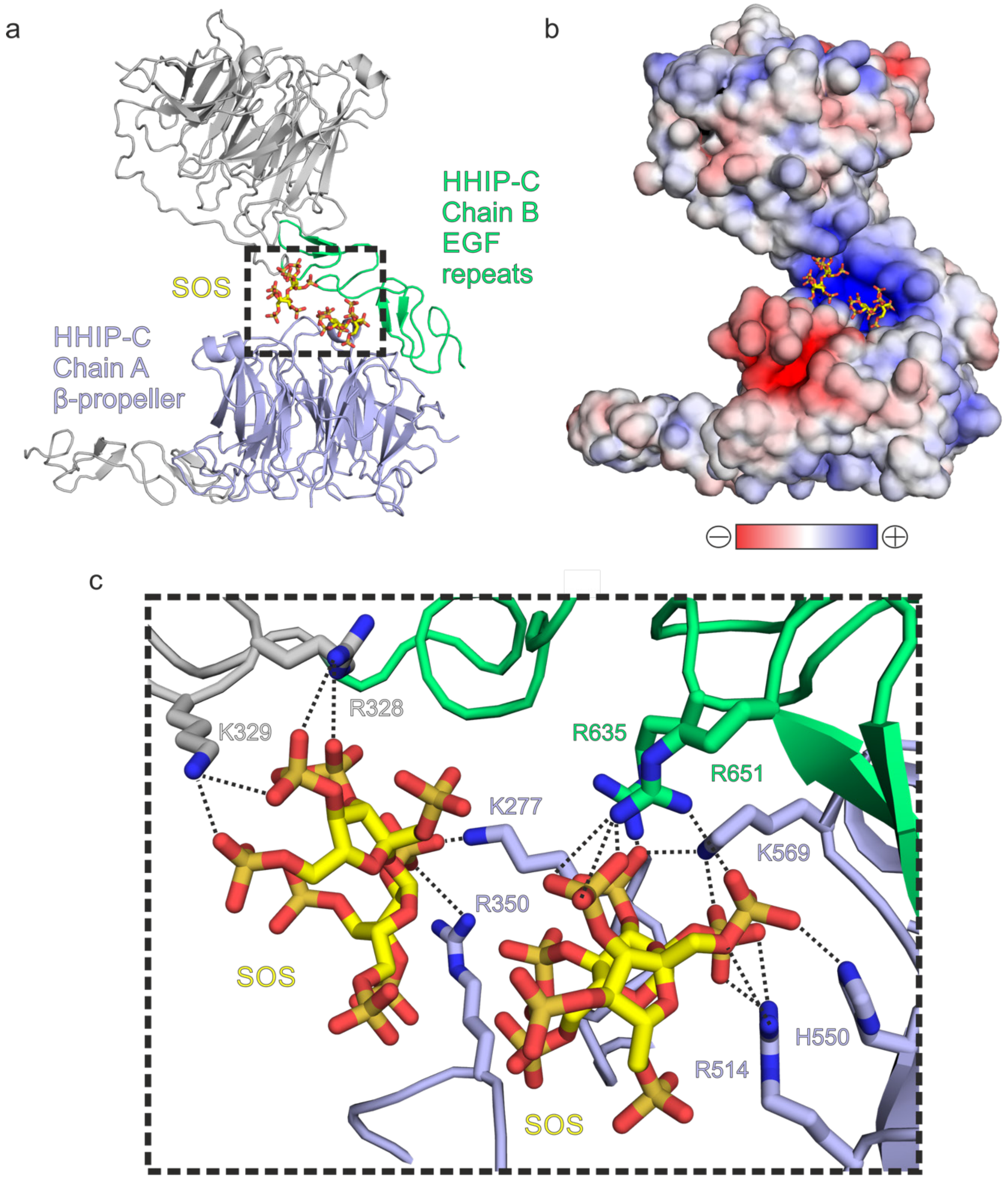

Supplementary Fig. 9. HHIP-C:SOS complex crystal structure. (a) Arrangement of two HHIP-C chains (a and b; cartoon representation) around two centrally coordinated SOS molecules (stick representation). Colour coding is as for Fig.4. **(b)** Electrostatic surface potential shown from red (-8 kT/e) to blue (+8 kT/e) with SOS molecules displayed. **(c)** Close-up view of the boxed region from (a). SOS molecules are labelled SOS1 and SOS2 and hydrogen bonds are displayed using dashed lines. Residues are coloured as in (a); two residues (R328, K329; grey) from a symmetry-related β-propeller region of HHIP-C chain B also contribute hydrogen-bonding interactions with SOS2.

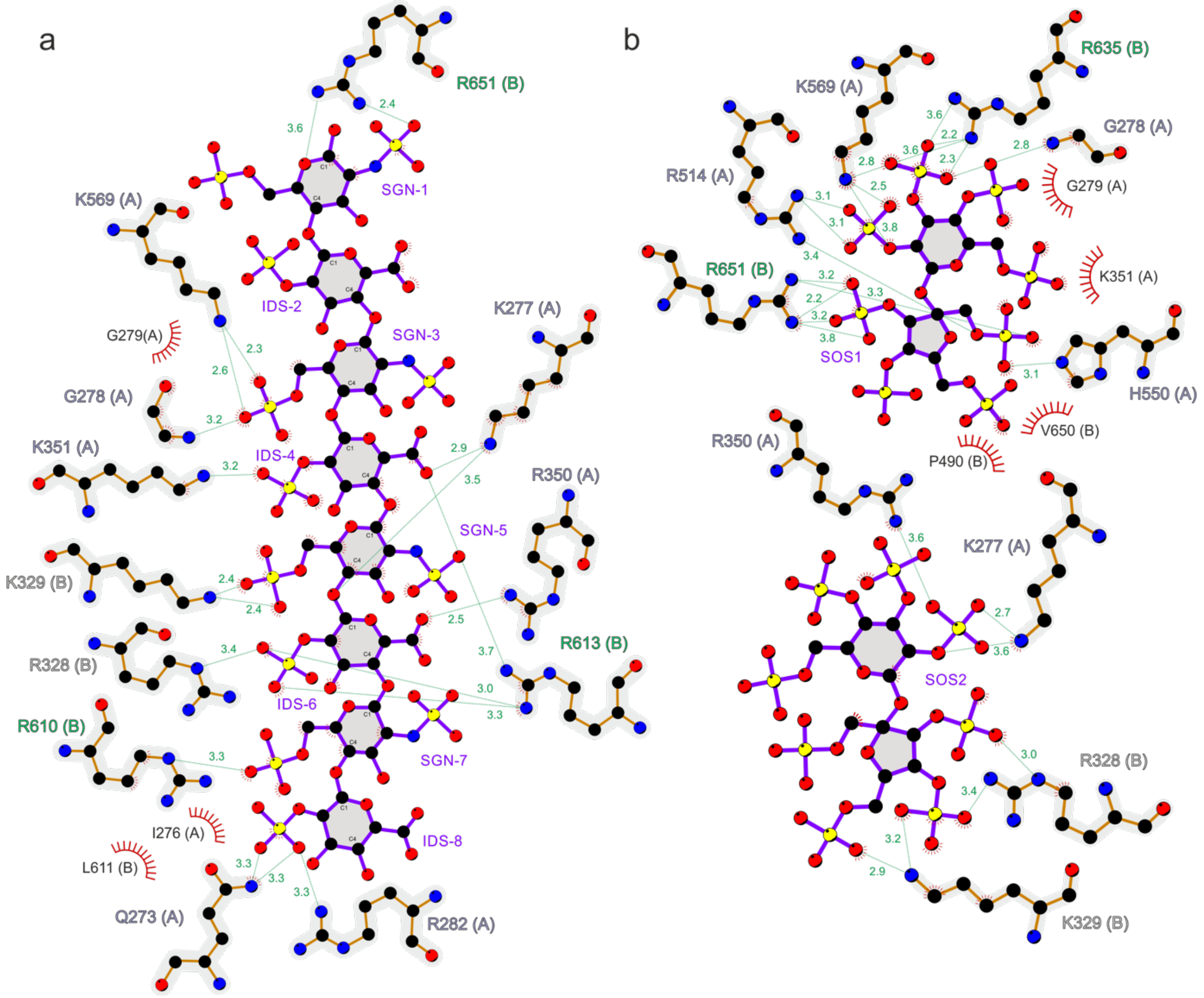

Supplementary Fig. 10. Schematic of HHIP-C:GAG interactions. Residues contacting heparin **(a)** and SOS **(b)** are displayed. Atoms are coloured as follows: N, blue; O, red; S, yellow; C, black. Covalent bonds are coloured violet (GAG) and orange (HHIP-C), and brackets after amino acids correspond to the HHIP-C chains as designated in Fig. 3a and Supplementary Fig. 7. Hydrogen bonds are displayed as green dashed lines and labelled with lengths in Ångstrom. Red 'eyelashes' correspond to hydrophobic interactions. SGN, O2-Sulphoiduronic acid; IDS, N,O6-disulphoglucosamine. Adapted from PDBsum [20] and PISA [21].

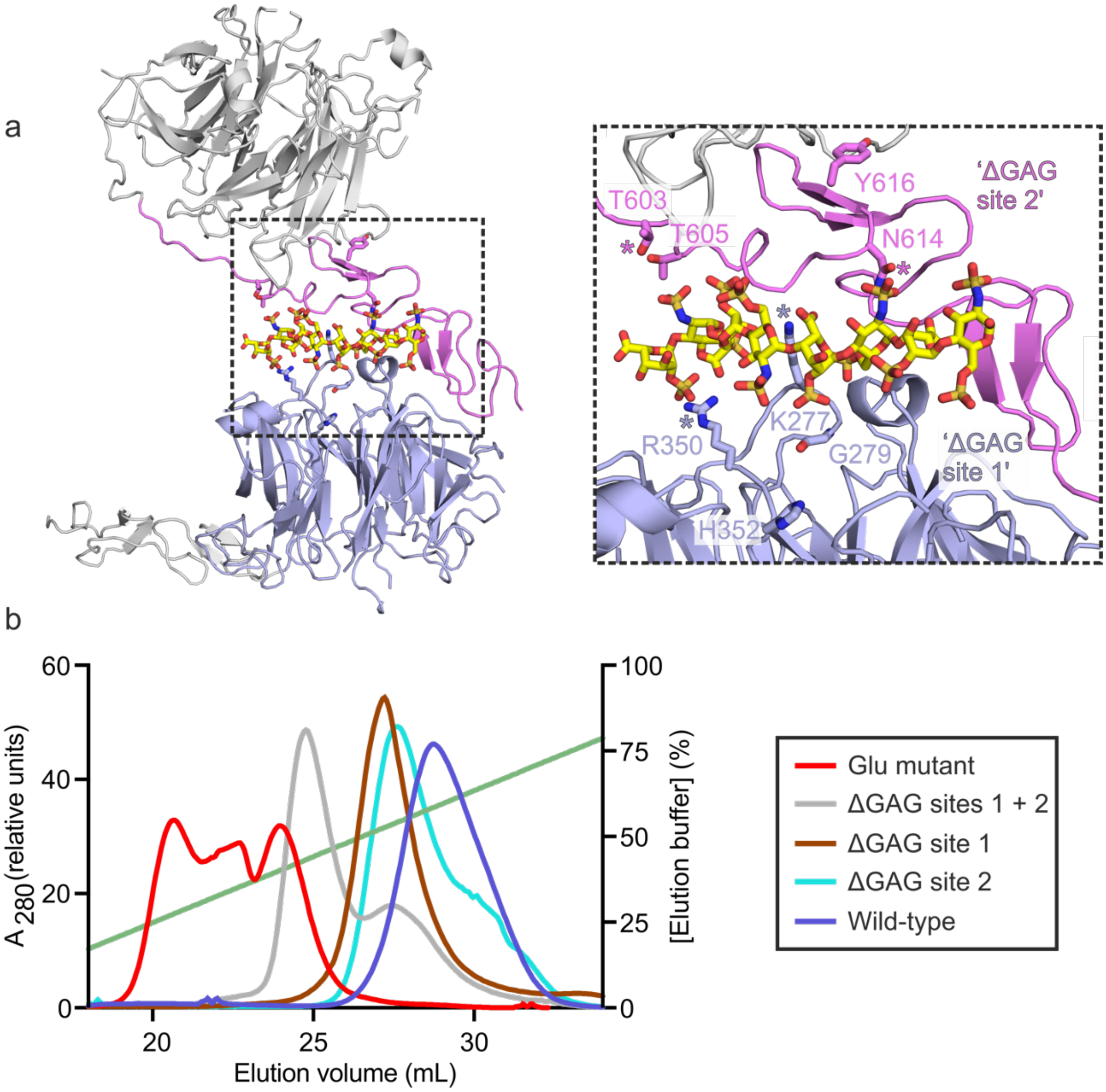

Supplementary Fig. 11. Heparin affinity chromatography of HHIP-C constructs. (a) Rationale for the design of ΔGAG site mutants. N-linked sugar consensus sequences (N-X-T/S) were inserted into the two sites of GAG interaction (EGF repeats: pink; β-propeller: light blue). As shown in the zoomed-in panel, two N-glycans were inserted into each site (mutations: site 1 – K277N/G279N/R350N/H352T; site 2 – T603N/Y616T). The N-linked residue produced after mutation is displayed using colour-coded asterisks. **(b)** The elution of HHIP-C constructs was analysed using a heparin-binding assay. Elution was followed by absorption at 280 nm (colour-coded, inset) with the elution gradient displayed as a green trace. Wild-type HHIP-C (purple) binds with highest affinity, followed by the individual GAG site mutants (cyan and brown) and the dual GAG site mutant (grey). The HHIP-C Glu mutant (red) displays the largest decrease in heparin binding affinity.

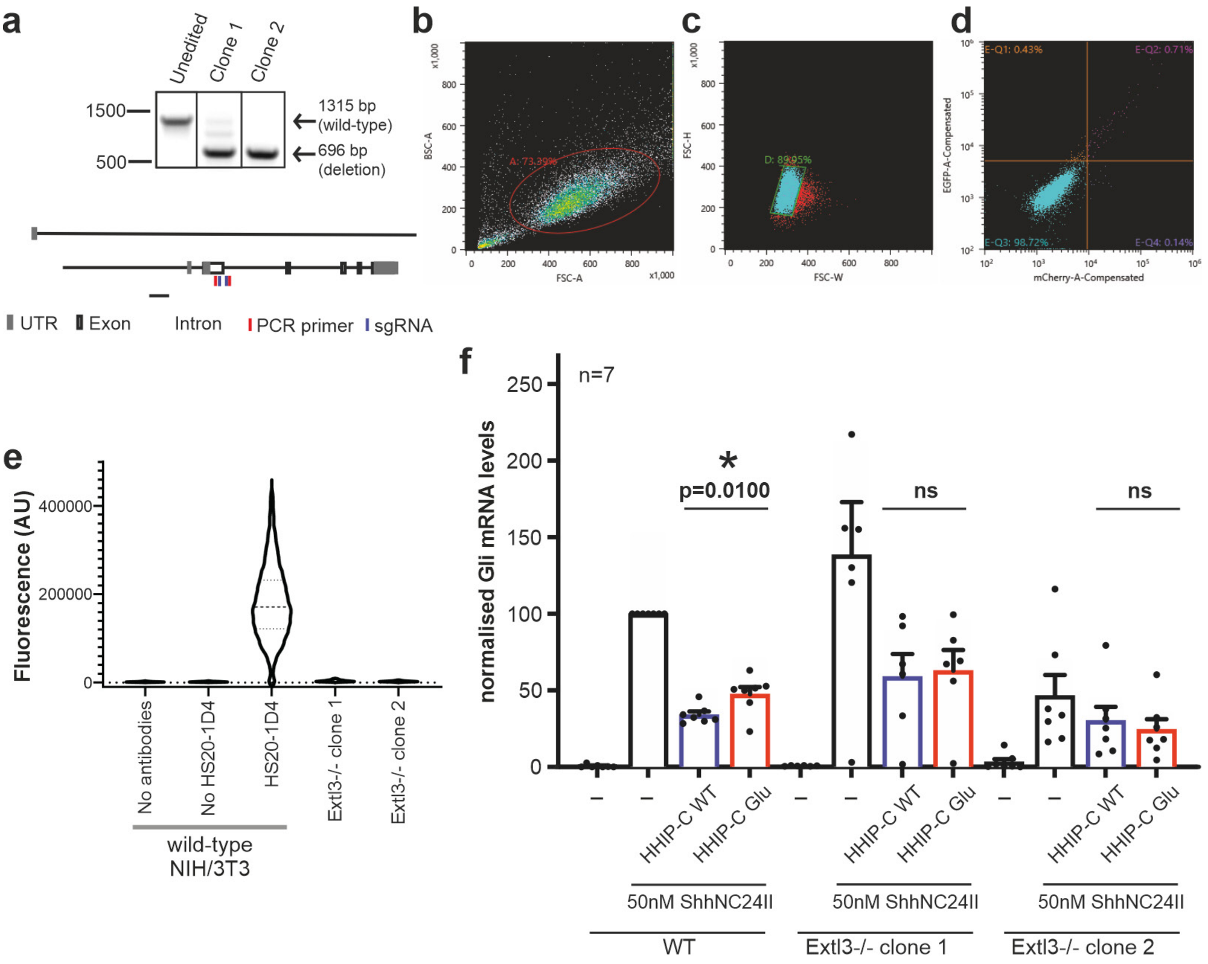

Supplementary Fig. 12. Quantification of HH pathway inhibition in HS deficient mouse 3T3 fibroblasts in an Extl3-/- background. (a) Generation of mouse NIH/3T3 *Extl3*$^{-/-}$ cells. Gel showing successful gene editing, revealed by deletion of a segment of genomic DNA between the two guides, as shown in the schematic below. **(b-d)** Plots show the gating strategy used to identify cells that were transfected with two plasmids, each carrying one of the two guides used in the double-cut CRISPR strategy to ablate Extl3 (see methods). One plasmid encodes GFP and the second encodes mCherry so that doubly transfected cells will be positive for both GFP and RFP. Gating based on forward and back scatter (**b**) (FSC and BSC respectively) was used to indentify live cells (~73% of the population). Gating based on FSC width (W) vs height (H) (**c**) was used to identify single cells (~90% of the population of live cells). Finally, gating based on EGFP fluorescence (Y-axis) and mCherry fluorescence (X-axis) (**d**) was used to find double transfected cells (0.71% of the single population from B in Quadrant 2 (Q2). Quadrants were set such that 0% of cells appeared in Q1, Q2, and Q4 in a mock-tranfected plate. All single cells isolated by this method were expanded and the knock-out of Extl3 confirmed by independent methods (see **Supplementary Fig. 12e**). **(e)** Cell surface HSPGs were measured in the indicated cell lines, including two clonal *Extl3*$^{-/-}$ cell lines, using staining with the anti-Heparan Sulfate HS20-1D4 antibody (see methods). Outliers eliminated using the ROUT method (Q=10%). **(f)** HH signalling assay to assess HHIP-C WT or HHIP-C Glu mutant inhibition of HH pathway activation in response to ShhN. Relative levels of Gli1 mRNA were quantified from 7 independent experiments, with statistical significance calculated using a two-tailed, paired t-test with p=0.01 for wildtype 3T3 cells, but no significant difference for two independent 3T3 Extl-/- clones.

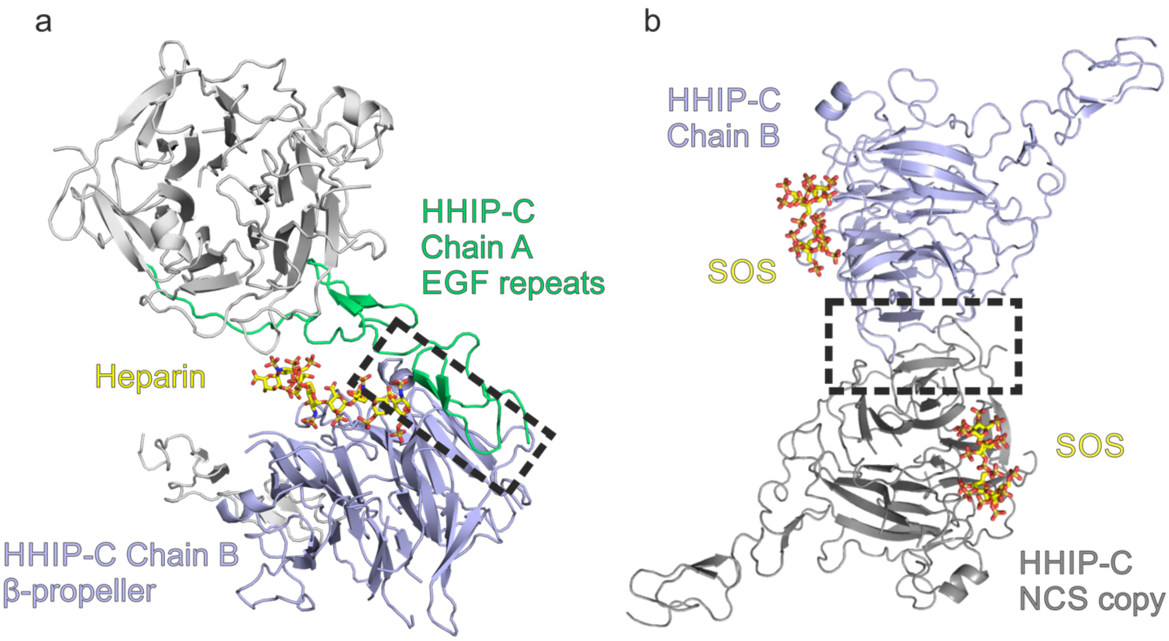

Supplementary Fig. 13. HHIP-C dimer interactions. **(a)** Anti-parallel 'head-to-tail' dimer. The HHIP-C:heparin complex crystal structure at 2.7 Å is displayed, with the protein backbone depicted in cartoon representation and the heparin molecule as sticks. A boxed region indicates the dimer interface (interface area = 603 $Å^2$; 5 hydrogen bonds, 55 van der Waals interactions); **(b)** 'Head-to-head' dimer. The asymmetric unit of the HHIP-C:SOS complex at 2.4 Å resolution (highest to date) is displayed, with the protein backbone depicted in cartoon representation and the SOS molecules as sticks. A boxed region indicates the dimer interface (dimer interfacial area = 981 $Å^2$; 5 hydrogen bonds, 88 van der Waals interactions).

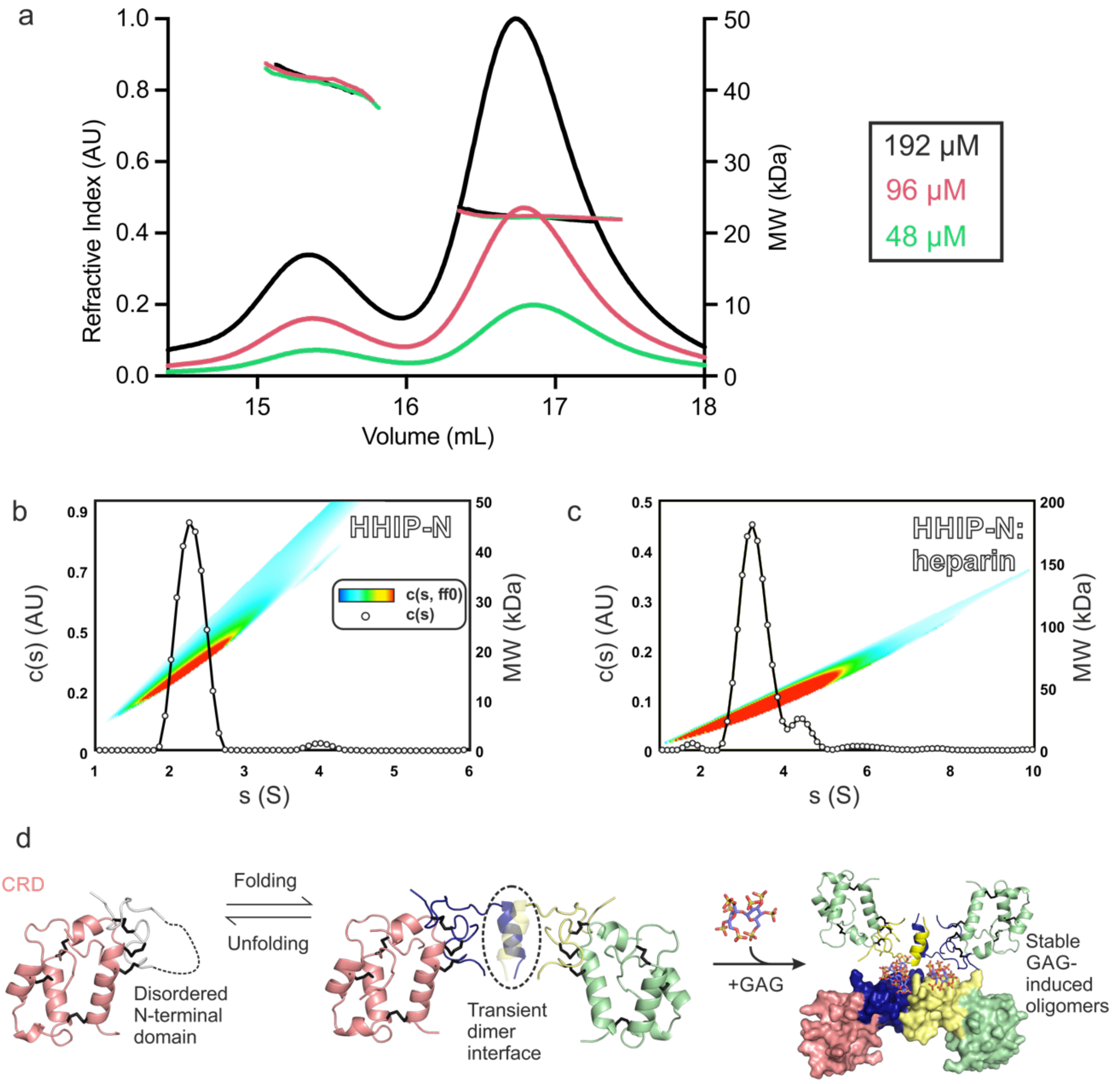

Supplementary Fig. 14. Analysis of the oligomeric behaviour of HHIP-N. **(a)** SEC-MALS analysis of HHIP-N. Eluted peaks are shown as solid lines corresponding to refractive index readings (Y-axis 1), whilst the corresponding calculated molecular weights (MW) are shown as dotted lines for each peak (Y-axis 2). Concentrations are colour-coded (inset). **(b-c)** AUC experiments of HHIP-N (b) and HHIP-N: heparin (30-mer) complex (c). **(d)** Model for HHIP-N clustering. HHIP-N monomer:dimer equilibrium exists due to folding and unfolding of the N-terminal domain. GAGs are able to stabilise the N-terminal domain and therefore the dimer interface, ultimately inducing higher-order oligomeric states via recruitment of HHIP-N dimers (right).

SUPPLEMENTARY REFERENCES

1 Riffel, N. *et al.* Atomic Resolution Structure of Moloney Murine Leukemia Virus Matrix Protein and Its Relationship to Other Retroviral Matrix Proteins. *Structure (London, England : 1993)* **10**, 1627-1636, doi:10.1016/S0969-2126(02)00896-1 (2002).

2 Stuart, D. I., Levine, M., Muirhead, H. & Stammers, D. K. Crystal structure of cat muscle pyruvate kinase at a resolution of 2.6 A. *Journal of molecular biology* **134**, 109-142, doi:10.1016/0022-2836(79)90416-9 (1979).

3 Felsenstein, J. PHYLIP: Phylogeny Inference Package. Version 3.2. Joel Felsenstein. **64**, 539-541, doi:10.1086/416571 (1989).

4 Janda, C. Y., Waghray, D., Levin, A. M., Thomas, C. & Garcia, K. C. Structural basis of Wnt recognition by Frizzled. *Science (New York, N.Y.)* **337**, 59-64, doi:10.1126/science.1222879 (2012).

5 Byrne, E. F. X. *et al.* Structural basis of Smoothened regulation by its extracellular domains. *Nature*, doi:10.1038/nature18934 (2016).

6 Dann, C. E. *et al.* Insights into Wnt binding and signalling from the structures of two Frizzled cysteine-rich domains. *Nature* **412**, 86-90, doi:10.1038/35083601 (2001).

7 Stiegler, A. L., Burden, S. J. & Hubbard, S. R. Crystal structure of the frizzled-like cysteine-rich domain of the receptor tyrosine kinase MuSK. *Journal of molecular biology* **393**, 1-9, doi:10.1016/j.jmb.2009.07.091 (2009).

8 Kwon, H. J. *et al.* Structure of N-terminal domain of NPC1 reveals distinct subdomains for binding and transfer of cholesterol. *Cell* **137**, 1213-1224, doi:10.1016/j.cell.2009.03.049 (2009).

9 Monaco, H. L. Crystal structure of chicken riboflavin-binding protein. *The EMBO journal* **16**, 1475-1483, doi:10.1093/emboj/16.7.1475 (1997).

10 Chen, C. *et al.* Structural basis for molecular recognition of folic acid by folate receptors. *Nature* **500**, 486-489, doi:10.1038/nature12327 (2013).

11 Wibowo, A. S. *et al.* Structures of human folate receptors reveal biological trafficking states and diversity in folate and antifolate recognition. *Proceedings of the National Academy of Sciences of the United States of America* **110**, 15180-15188, doi:10.1073/pnas.1308827110 (2013).

12 Han, L. *et al.* Divergent evolution of vitamin B9 binding underlies Juno-mediated adhesion of mammalian gametes. *Current biology: CB* **26**, R100-101, doi:10.1016/j.cub.2015.12.034 (2016).

13 Rudolf, A. F. *et al.* The morphogen Sonic hedgehog inhibits its receptor Patched by a pincer grasp mechanism. *Nature chemical biology* **15**, 975-982, doi:10.1038/s41589-019-0370-y (2019).

14 Luque, I. & Freire, E. Structure-based prediction of binding affinities and molecular design of peptide ligands. *Methods Enzymol* **295**, 100-127, doi:10.1016/s0076-6879(98)95037-6 (1998).

15 Bishop, B. *et al.* Structural insights into hedgehog ligand sequestration by the human hedgehog-interacting protein HHIP. *Nature structural & molecular biology* **16**, 698-703, doi:10.1038/nsmb.1607 (2009).

16 Bosanac, I. *et al.* The structure of SHH in complex with HHIP reveals a recognition role for the Shh pseudo active site in signaling. *Nature structural & molecular biology* **16**, 691-697, doi:10.1038/nsmb.1632 (2009).

17 Healey, E. G. *et al.* Repulsive guidance molecule is a structural bridge between neogenin and bone morphogenetic protein. *Nature structural & molecular biology* **22**, 458-465, doi:10.1038/nsmb.3016 (2015).

18 Molday, R. S. & MacKenzie, D. Monoclonal antibodies to rhodopsin: characterization, cross-reactivity, and application as structural probes. *Biochemistry* **22**, 653-660 (1983).

19 Chazotte, B. Labeling nuclear DNA with hoechst 33342. *Cold Spring Harbor protocols* **2011**, pdb.prot5557 (2011).

20 Laskowski, R. A. PDBsum: summaries and analyses of PDB structures. *Nucleic acids research* **29**, 221-222 (2001).

21 Krissinel, E. & Henrick, K. Inference of Macromolecular Assemblies from Crystalline State. *Journal of molecular biology* **372**, 774-797, doi:10.1016/j.jmb.2007.05.022 (2007).
