# Supplementary material for: Hedgehog-Interacting Protein is a multimodal antagonist of Hedgehog signalling
Source: Nat Commun. 2021 Dec 9;12:7171. doi: 10.1038/s41467-021-27475-2 (PMC8660895; doi:10.1038/s41467-021-27475-2)
Supplement: Supplementary file 2 — Reporting Summary [file 41467_2021_27475_MOESM2_ESM.pdf]

nature research

Corresponding author(s): Prof. Christian Siebold

Last updated by author(s): Jun 6, 2021

Reporting Summary

Nature Research wishes to improve the reproducibility of the work that we publish. This form provides structure for consistency and transparency in reporting. For further information on Nature Research policies, see our Editorial Policies and the Editorial Policy Checklist.

Statistics

For all statistical analyses, confirm that the following items are present in the figure legend, table legend, main text, or Methods section.

n/a	Confirmed	
☐	☒	The exact sample size (*n*) for each experimental group/condition, given as a discrete number and unit of measurement
☐	☒	A statement on whether measurements were taken from distinct samples or whether the same sample was measured repeatedly
☐	☒	The statistical test(s) used AND whether they are one- or two-sided<br>*Only common tests should be described solely by name; describe more complex techniques in the Methods section.*
☒	☐	A description of all covariates tested
☒	☐	A description of any assumptions or corrections, such as tests of normality and adjustment for multiple comparisons
☐	☒	A full description of the statistical parameters including central tendency (e.g. means) or other basic estimates (e.g. regression coefficient) AND variation (e.g. standard deviation) or associated estimates of uncertainty (e.g. confidence intervals)
☐	☒	For null hypothesis testing, the test statistic (e.g. *F*, *t*, *r*) with confidence intervals, effect sizes, degrees of freedom and *P* value noted<br>*Give P values as exact values whenever suitable.*
☒	☐	For Bayesian analysis, information on the choice of priors and Markov chain Monte Carlo settings
☒	☐	For hierarchical and complex designs, identification of the appropriate level for tests and full reporting of outcomes
☒	☐	Estimates of effect sizes (e.g. Cohen's *d*, Pearson's *r*), indicating how they were calculated

Our web collection on statistics for biologists contains articles on many of the points above.

Software and code

Policy information about availability of computer code

Data collection	CHEMIDOC MP IMAGING SYSTEM (Bio-rad) - SDS PAGE gels and Western blot imaging.<br>LiCor Odyssey-- Quantitative Western Blotting for calculating concentrations of SHH and variants.<br>QuantStudio 3-- Quantitative RT-PCR Studies.<br>MicroCal PEAQ-ITC v1.21 (Malvern) - ITC data acquisition.<br>BIAcore T2000 (GE Healthcare) - SPR data acquisition.<br>Beckman Optima XL-1 (Beckman Instruments) - AUC data acquisition.<br>Wyatt Dawn HELEOS-II MALS detector and Wyatt Optilab rEX refractive index monitor - MALS data acquisition.
Data analysis	GraphPad Prism v8 - Analysis and statistics for the HH signalling assays (https://www.graphpad.com/scientific-software/prism/).<br>SCRUBBER2 and GraphPad Prism 6.04 - SPR data analysis (http://www.biologic.com.au/scrubber.html and https://www.graphpad.com/scientific-software/prism/).<br>NITPIC (1.3.0), SEDPHAT (15.2b) and GUSSI (1.4.2) - ITC data analysis (http://biophysics.swmed.edu/MBR/software.html, http://www.analyticalultracentrifugation.com/sedphat/ and http://biophysics.swmed.edu/MBR/software.html).<br>MicroCal PEAQ-ITC (1.0.0.1259) (Malvern) - ITC data analysis.<br>Fiji (ImageJ 2.0.0-rc-2) - Analysis of microscopy images (https://imagej.net/software/fiji/downloads).<br>SEDFIT (14.4f) - AUC data analysis (http://www.analyticalultracentrifugation.com/default.htm).<br>ASTRA software (Wyatt Technology) - MALS data analysis (https://www.wyatt.com/)<br>XIA2 (Diamond Light Source) - automated reduction of X-ray diffraction data (https://www.diamond.ac.uk/Instruments/Mx/Common/Common-Manual/Data-Analysis/Automated-Software-Pipeline/Immediately-Following-Data-Collection.html).<br>DIALS - Diffraction Integration for Advanced Light Sources (https://dials.github.io/).<br>SCALA - analysis (scaling) of X-ray diffraction data (https://www.mrc-lmb.cam.ac.uk/harry/pre/scala.html).<br>PHASER (2.7.17) - molecular replacement (http://www.phaser.cimr.cam.ac.uk/index.php/Phaser_Crystallographic_Software).<br>XDS - analysis (indexing and integration) of X-ray diffraction data (http://xds.mpimf-heidelberg.mpg.de).<br>AIMLESS - analysis (scaling) of X-ray diffraction data (http://www.ccp4.ac.uk/html/aimless.html).

CCP4 (7.0.023)- Software for Macromolecular Crystallography (http://www.ccp4.ac.uk/).
PHENIX (1.13rc2_2986) - analysis, validation and manipulation of X-ray diffraction data (https://www.phenix-online.org).
COOT (0.8.9.1) - building and validation of atomic models (https://www2.mrc-lmb.cam.ac.uk/personal/pemsley/coot/).
MOLPROBITY - validation of structures (http://molprobity.biochem.duke.edu).
PRIVATEER - conformational validation of sugar models (http://www.ccp4.ac.uk/html/privateer.html).
WWPDB VALIDATION SERVICE - validation of structures (https://validate-rcsb-1.wwpdb.org).
PYMOL (Schrodinger, LLC, 2.1) - molecular visualization system (https://pymol.org/2/).
UCSF CHIMERA - extendible molecular modeling system (https://www.cgl.ucsf.edu/chimera/).
ALINE - alignment of amino acid sequences (https://bondxray.org/software/aline.html).
PHYLIP - structure-based evolutionary analysis (http://evolution.genetics.washington.edu/phylip.html).
APBS - calculation of electrostatic and solvation properties for proteins (http://www.poissonboltzmann.org/).
PDBSUM - analysis of protein secondary structure, protein-protein interfaces (www.ebi.ac.uk/pdbsum).
PDBEPISA - analysis of macromolecular interfaces (http://www.ebi.ac.uk/pdbe/pisa/).
SHELX Version 0.4e (http://webapps.embl-hamburg.de/hkl2map/).
REFMAC5 (http://www.ccp4.ac.uk/download).
AUTOBUSTER version 5 (https://www.globalphasing.com/buster/).
BUSTER 2.10.3

For manuscripts utilizing custom algorithms or software that are central to the research but not yet described in published literature, software must be made available to editors and reviewers. We strongly encourage code deposition in a community repository (e.g. GitHub). See the Nature Research guidelines for submitting code & software for further information.

Data

Policy information about availability of data

All manuscripts must include a data availability statement. This statement should provide the following information, where applicable:

- Accession codes, unique identifiers, or web links for publicly available datasets
- A list of figures that have associated raw data
- A description of any restrictions on data availability

The PDB files and corresponding MTZ files for HHIP-N:SOS, HHIP-N apo, HHIP-C:heparin, HHIP-C:SOS have been deposited in the Protein Data Bank (PDB) (www.rcsb.org) under accession numbers 7PGK, 7PGL, 7PGM and 7PGN, respectively.

Field-specific reporting

Please select the one below that is the best fit for your research. If you are not sure, read the appropriate sections before making your selection.

☒ Life sciences ☐ Behavioural & social sciences ☐ Ecological, evolutionary & environmental sciences

For a reference copy of the document with all sections, see nature.com/documents/nr-reporting-summary-flat.pdf

Life sciences study design

All studies must disclose on these points even when the disclosure is negative.

Sample size	Sample sizes were estimated on the basis of previous studies using similar methods and analyses that are widely published. For SHH dose-response curves (Figures 2 and 5, and Supplementary Figure 12), no sample size calculation was performed; sample size was chosen such that statistical significance could be confidently established.
Data exclusions	No data was excluded.
Replication	All attempts to replicate data were successful.
Randomization	Randomization and blinding were not used in our study since none of the experiments relied on subjective assessment (all were measured using automated machines without human intervention and so 'blind' to the machine) nor did any involve trials with animal or human subjects. The same individual set-up and analyzed each experiment. The outcome of each experiment (e.g. Gli1 mRNA levels) was assessed using a quantitative assay (not a subjective assessment).
Blinding	All data collection for all assays used automated systems to measure output from different experiments, all experiments were effectively 'blind' as the automated analysis did not know what each sample was. The outcome of each experiment (e.g. Gli1 mRNA levels) was assessed using a quantitative assay (not a subjective assessment).

Reporting for specific materials, systems and methods

We require information from authors about some types of materials, experimental systems and methods used in many studies. Here, indicate whether each material, system or method listed is relevant to your study. If you are not sure if a list item applies to your research, read the appropriate section before selecting a response.

Materials & experimental systems

n/a	Involved in the study
☐	☒ Antibodies
☐	☒ Eukaryotic cell lines
☒	☐ Palaeontology and archaeology
☒	☐ Animals and other organisms
☒	☐ Human research participants
☒	☐ Clinical data
☒	☐ Dual use research of concern

Methods

n/a	Involved in the study
☒	☐ ChIP-seq
☐	☒ Flow cytometry
☒	☐ MRI-based neuroimaging

Antibodies

Antibodies used

Penta-His Antibody, BSA free (Qiagen. cat. no. 34660, lot. no. 160017634).
HA -Tag Monoclonal Antibody (2-2.2.14)(Catalog # 26183, Thermo Fisher).
Anti-mouse IgG (Fc specific) - Peroxidase polyclonal goat antibody (Sigma, cat. no. A0168, lot. no. 068M4764V) (used 1:5000).
Goat anti-Mouse IgG (H+L) Highly Cross-Adsorbed Secondary Antibody, Alexa Fluor 633 (Thermofisher cat. no. A-21052) (used 1:5000).
Rho 1D4 mouse monoclonal (University of British Columbia: https://ubc.flintbox.com/technologies/0f1ef64b-fa5d-4a58-9003-3e01f6f672a6)
Donkey anti-mouse antibody coupled to Alexa Fluor 594 (1:500; ThermoFisher Scientific Cat# A-21203)

Validation

Antibodies were validated by the manufacturers:

Anti-penta His antibody: According to the manufacturer's website (https://www.qiagen.com/gb/products/discovery-and-translational-research/protein-purification/tagged-protein-expression-purification-detection/penta-his-antibody-bsa-free/#productdetails) this antibody has a sensitivity of 50 pg for chemiluminescent western blots detecting N-, C- and internal His tags and has been cited in over 100 prior publications. We have used this antibody to detect His tagged proteins for over 2000 different recombinant proteins made in HEK293T and HEK293S (GnTI-) cells. To validate specificity, we have run the same samples with different tags (e.g. Rho 1D4) and not had any cross-reactivity, we also run negative controls to negate detection of a non-specific band from serum-containing media and ensure it is not mistaken for the protein being studied.

Goat anti-mouse IgG secondary antibody: According to the manufacturer's website (https://www.sigmaaldrich.com/catalog/product/sigma/a0168?lang=en&region=GB), there is no observed cross-reactivity in westerns when the primary antibody is not applied, a result that we have also tested. From the manufacturer's website, this antibody has been cited in 94 prior publications as well as many more from this and other laboratories.

Goat anti-Mouse IgG (H+L) Highly Cross-Adsorbed Secondary Antibody, Alexa Fluor 633 labeled. This antibody was cited 41 times (as stated on the manufacturerer's website: https://www.thermofisher.com/antibody/product/Goat-anti-Mouse-IgG-H-L-Highly-Cross-Adsorbed-Secondary-Antibody-Polyclonal/A-21052) and no cross-reactivity was observed.

Rho 1D4: Rho 1D4 specifically binds to the C- terminal epitope -T-E-T-S-Q-V-A-P-A- (COOH) of rhodopsin antibody has a sensitivity of >50 pg for chemiluminescent western blots detecting C-terminal Rho-1D4 tag tags and has been cited in over 100 prior publications. We have used this antibody to detect His tagged proteins for over 1000 different recombinant proteins made in HEK293T and HEK293S (GnTI-) cells. To validate specificity, we have run the same samples with different tags (e.g. hexahis, double strep tag) and not had any cross-reactivity, we also run negative controls to negate detection of a non-specific band from serum-containing media and ensure it is not mistaken for the protein being studied. (references supplied by UBC):
-RS Molday et al. Monoclonal Antibodies to Rhodopsin: Characterization, Cross-Reactivity, and Application as Structural Probes. Biochemistry 1983 (22) 653-660. DOI: 10.1021/bi00272a020.
-DD Oprian et al. Expression of a Synthetic Bovine Rhodopsin Gene in Monkey Kidney Cells. Procl. Natl. Acad. Sci. 1987 (84) 8874-8878. DOI: 10.1073/pnas.84.24.8874.
-RS Hodgest et al. Antigen-Antibody Interaction. Synthetic peptides Define Linear Antigen Deterinants Recognized by Monoclonal Antibodies Directed to the Cytoplasmic Carboxyl Terminus of Rhodopsin. J. Biol. Chem. 1988 (263) 11768-11775. DOI:jbc.263.24.11768.

Eukaryotic cell lines

Policy information about cell lines

Cell line source(s)

- HEK293T (ATCC CRL-3216)
- NIH/3T3 (ATCC CRL-1658)
- PANC-1 (ATCC CRL-1469)
- EXTL3-/- cells are a clonal cell line generated by CRISPR editing using the NIH/3T3 (ATCC CRL-1658) cells purchased from ATCC. The generation and authentication of this cell line is described in the methods. Briefly, editing of the Extl3 locus was confirmed by genomic PCR and loss of cell-surface heparin sulfate expression. No further authentication was performed.

Authentication

HEK293T (ATCC CRL-3216), NIH/3T3 (ATCC CRL-1658) and PANC-1 (ATCC CRL-1469) cell lines were authenticated by the suppliers listed above and used at low passage; they were not were not re-authenticated in our laboratories.

Mycoplasma contamination

All cell lines were tested for mycoplasma contamination and were negative.

Commonly misidentified lines (See ICLAC register)

No commonly misidentified cell lines were used.

Flow Cytometry

Plots

Confirm that:

☒ The axis labels state the marker and fluorochrome used (e.g. CD4-FITC).

☒ The axis scales are clearly visible. Include numbers along axes only for bottom left plot of group (a 'group' is an analysis of identical markers).

☒ All plots are contour plots with outliers or pseudocolor plots.

☒ A numerical value for number of cells or percentage (with statistics) is provided.

Methodology

Sample preparation

See methods. NIH/3T3 cells were transfected with CRISPR plasmids. Four days after transfection, cells were trypsinized and subjected to FACS to select single cells that expressed both GFP and mCherry. Clonal cell lines were then tested to ensure excision of a DNA segment between the two cut sites.

Instrument

Sony SH800S Cell Sorter

Software

Sony SH800S system software was used.

Cell population abundance

The abundances of the cell populations that were selected in each gate are shown in Supplementary Figure X. 0.71% of the cells expressed both GFP and RFP and hence were propagated as single cell clones to test for ablation of Extl3.

Gating strategy

Our gating strategy is described in Supplementary Figure X. Please note that there is no data in the paper that uses flow cytometry. FACS was used only to isolate single cells transfected with the CRISPR plasmids for genome editing (which was confirmed independently).

☒ Tick this box to confirm that a figure exemplifying the gating strategy is provided in the Supplementary Information.
